# Protamine 1 as a secreted colorectal cancer-specific antigen facilitating G1/S phase transition under nutrient stress conditions

**DOI:** 10.1007/s13402-022-00754-w

**Published:** 2023-01-03

**Authors:** Shengnan Ren, Dingquan Yang, Yongli Dong, Weidong Ni, Meiqi Wang, Lei Xing, Tong Liu, Wenjia Hou, Weixuan Sun, Haolong Zhang, Zhentao Yu, Yi Liu, Jingrui Cao, Hongbo Yan, Ye Feng, Xuedong Fang, Quan Wang, Fangfang Chen

**Affiliations:** 1 Key Laboratory of Zoonoses Research, Ministry of Education, Nanomedicine and Translational Research Center, China-Japan Union Hospital of Jilin University, Changchun, China; 2 Department of Gastrointestinal, Colorectal and Anal Surgery, China-Japan Union Hospital of Jilin University, Changchun, China; 3 Department of Gastrointestinal Surgery, Heze Municipal Hospital, Heze, China; 4 Department of Burn Surgery, The First Hospital of Naval Medical University, Shanghai, China; 5 Department of Gastrointestinal Surgery, The First Hospital of Jilin University, Changchun, Jilin, China

**Keywords:** Cancer testis antigen, PRM1, Metabolism rewiring, Cell proliferation, Serum deficiency

## Abstract

**Purpose:**

Cancer testis antigens (CTAs) are optimal tumor diagnostic markers and involved in carcinogenesis. However, colorectal cancer (CRC) related CTAs are less reported with impressive diagnostic capability or relevance with tumor metabolism rewiring. Herein, we demonstrated CRC-related CTA, Protamine 1 (PRM1), as a promising diagnostic marker and involved in regulation of cellular growth under nutrient deficiency.

**Methods:**

Transcriptomics of five paired CRC tissues was used to screen CRC-related CTAs. Capability of PRM1 to distinguish CRC was studied by detection of clinical samples through enzyme linked immunosorbent assay (ELISA). Cellular functions were investigated in CRC cell lines through in vivo and in vitro assays.

**Results:**

By RNA-seq and detection in 824 clinical samples from two centers, PRM1 expression were upregulated in CRC tissues and patients` serum. Serum PRM1 showed impressive accuracy to diagnose CRC from healthy controls and benign gastrointestinal disease patients, particularly more sensitive for early-staged CRC. Furthermore, we reported that when cells were cultured in serum-reduced medium, PRM1 secretion was upregulated, and secreted PRM1 promoted CRC growth in culture and in mice. Additionally, G1/S phase transition of CRC cells was facilitated by PRM1 protein supplementation and overexpression via activation of PI3K/AKT/mTOR pathway in serum deficient medium.

**Conclusions:**

In general, our research presented PRM1 as a specific CRC antigen and illustrated the importance of PRM1 in CRC metabolism rewiring. The new vulnerability of CRC cells was also provided with the potential to be targeted in future.

**Graphical abstract:**

Diagnostic value and grow factor-like biofunction of PRM1 A represents the secretion process of PRM1 regulated by nutrient deficiency. B represents activation of PI3K/AKT/mTOR pathway of secreted PRM1.

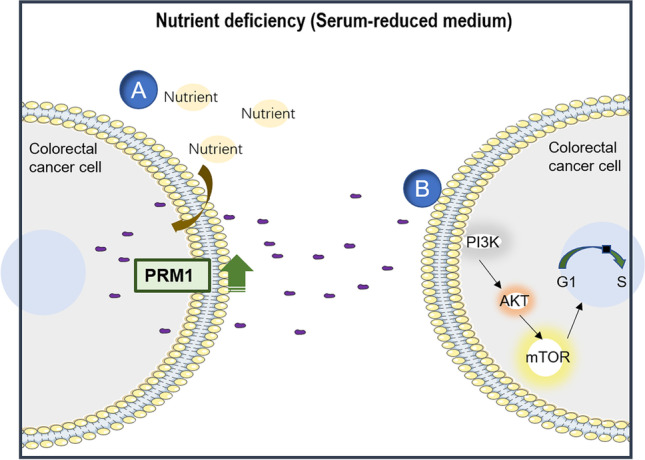

**Supplementary Information:**

The online version contains supplementary material available at 10.1007/s13402-022-00754-w.

## Introduction

Cancer-testis antigens (CTAs), are tumor associated antigens which have restricted expression patterns primarily in male testis, placenta, and some malignancies. Possessing certain immunogenicity, they are now considered as optimal target library for tumor diagnosis and immunotherapy [1]. Many CTAs induce humoral or cellular immune response in patients, rendering opportunities for cancer immunotherapy including T-cell receptors (TCRs), CAR T cell, antibody-based therapy, and tumor therapeutic vaccines [2–5]. Functionally, CTAs are also involved in regulating many cellular processes during tumorigenesis: transcriptional regulation, mitotic fidelity, and protein degradation [6]. Colorectal cancer (CRC) is one of the most common malignancies worldwide, and several CTAs have been identified in CRC. However, few was reported with high expression frequency or diagnostic accuracy. Of all CRC-related CTAs, SPAG9 mRNA (Sperm associated antigen 9) and AKAP4 mRNA (A-kinase anchoring protein 4) were detected in 66% and 44% of 62 CRC tissues, respectively [7]. As for protein expression level, PLAC1 (placenta enriched 1) and MAGE-D4 were expressed in 56.7% (55/97) and 70% (21/30) CRC tissues [8, 9]. In that case, exploration of more CRC-related CTAs is still urgently needed.

During development and progression of malignancy, metabolism rewiring is critical to support the demand of uncontrolled proliferation and metastasis [10]. Several metabolic pathways are remodeled by autophagy and mitophagy in cancer cells to further sustain cell growth and anoikis, or develop treatment resistance [11]. Glycolysis is the most famous metabolism adaptation of tumor cells to generate lactate which is a basic metabolic molecular in many processes [12, 13]. Alterations in oxygen supply and nutrient composition in tumor microenvironment also leads to metabolism reprogramming [14–16]. CRC is a disease developed through multi processes, and several genetic alterations happen during the initiation and progression of colorectal tumors. Wnt, K-ras and p53 are well established drivers of CRC which influence cellular metabolism status in the process of tumorigenesis, and offers several therapeutic targets for CRC [11]. Therefore, understanding how cancer cells overcome metabolic challenges to sustain survival and progression is practical to develop novel options for CRC treatment.

In this study, we started with high throughput transcriptomic analysis of five paired CRC tissues, and reported nine CTAs abnormally expressed in CRC tissues (seven upregulated, two downregulated). Protamine 1 (PRM1, also known as CT94.1) was among the seven upregulated CRC-related CTAs, and less reported in literature. We investigated the expression pattern and diagnostic accuracy of PRM1 by detection of 824 clinical samples from two centers. Besides, the biofunctions and underlying cellular mechanism were well illustrated that PRM1 is involved in tumor metabolism rewiring under nutrition deficient conditions. Induction of PRM1 expression functioned in a growth factor-like manner to support CRC growth in culture and in mice, and specific antibody or knockdown of PRM1 expression exerted therapeutic effect on CRC.

## Materials and methods

### Patient and clinical samples

Tissue and serum samples from patients pathologically diagnosed with CRC (90 paired fresh frozen CRC tissues, 128 paraffin embedded CRC sections, and 218 serum samples), benign gastrointestinal disease (two fresh frozen colon epithelial tissues and 82 serum samples), and serum samples from healthy controls were obtained from China-Japan Union Hospital of Jilin University (Changchun, China) and the First Hospital of Jilin University (Changchun, China). Serum samples from CRC and benign gastrointestinal disease patients were collected before operation. Benign gastrointestinal diseases include gastric and colorectal adenoma or polyp, inflammatory bowel disease (ulcerative colitis and Crohn's disease), gastrointestinal benign tumors, acute appendicitis, perianal abscess, internal hemorrhoids and so on. Three-year survival information of patients in test cohort was followed-up from the date of surgery to the follow-up deadline, or date of death.

All experiments were approved by Ethics Review Committee of China-Japan Union Hospital of Jilin University and The First Hospital of Jilin University. Informed consent was obtained from participants, according to the committee’s regulations.

### Transcriptome high-throughput sequencing and bioinformatics analysis

RNA sequencing of five paired CRC tissues were performed by Cloud-Seq Biotech (Shanghai, China). The Database for Annotation, Visualization, and Integrated Discovery (DAVID) bioinformatics tool for KEGG pathway enrichment analysis and Gene Ontology 2 were applied to determine the roles that these differentially expressed mRNAs played in gene ontology (GO) terms of biological pathways.

### Immunohistochemical staining

Sections were incubated with antibodies against PRM1 (Hup-1 N, 1:100 dilution, Briar Patch Biosciences, USA), EGFR (RMA-0804, Maixin, China), VEGF (MAB-0243, Maixin, China), p53 (MAB-0674, Maixin, China), and HER-2 (4B5, Roche, USA).

Two pathologists who did not possess knowledge of the clinical data examined and scored all tissue specimens. Briefly, the IHC staining was semi-quantitatively scored as—(negative: no or less than 5% positive cells), + (6–25% positive cells), +  + (26–50% positive cells) and +  +  + (more than 50% positive cells).

### Enzyme-linked immunosorbent assay for PRM1

Peripheral blood samples were collected into anticoagulant-free tubes at the time of diagnosis. Enzyme-linked immunosorbent assay (ELISA) kits (CUSABIO, http://www.cusabio.com/, China) was used to detect the concentrations of serum PRM1. In each experiment, sample dilute buffer was used as control for serum PRM1 detection. All samples were detected in triplicate.

### Real-time PCR

We used SybrGreen (Roche, USA) for quantitative real-time PCR (RT-PCR). Sequence information of primers was seen in Table S1. Gene expression was normalized to GAPDH expression by the 2^−∆∆CT^ method.

### Cell lines

Human mammary epithelial cell line (MCF-10A) and CRC cell line HCT116 were purchased from National experimental cell resource sharing platform (Beijing, China). DLD-1, RKO, SW480 and SW620 were kindly provided by Cell Bank/Stem Cell Bank, Chinese Academy of Sciences (Shanghai, China). Cell lines were cultured in the corresponding medium containing 10% fetal bovine serum (FBS).

### Cell proliferation assay, EdU, wound healing and Transwell assay

Cells were cultured in the medium containing 1% FBS for 4 h, and treated with PRM1 protein or antibody against PRM1. Cell Counting Kit 8 (Dojindo) was used to analyze cell viability. After incubating with CCK8 for 1 h, color intensity was measured on a microplate reader at 450 nm (Biotek, Gen 5). EdU assay was conducted using the BeyoClick™ EdU Cell Proliferation Kit with Alexa Fluor 488 (Beyotime, China), according to the manufacturer’s protocol. Pictures were taken with a fluorescence microscope (Olympics IX51, IX83; Leica DMI3000 B).

Wound healing and Transwell assay were used to detect the migration and invasion ability of CRC cells. The distance between edges was examined using an inverted fluorescence microscope (Olympics IX51).

### Immunoblotting

Tissues or cell lysates were separated by Tricine-SDS PAEG for PRM1, or SDS–polyacrylamide gel electrophoresis (PAGE) for other proteins, followed by incubation with primary antibodies at 4℃ overnight (Table S2). Then, membranes were incubated with secondary antibodies. Bands were visualized by Odyssey (LI-COR) or chemiluminescence (Tanon, China).

### Immunofluorescence

Cells were cultured in the medium containing different concentrations of FBS for 24 h. Cells were then fixed in 4% paraformaldehyde (PFA), permeabilized with 0.3% (v/v) Triton X-100, and blocked (1% BSA). Cells were then incubated with anti-PRM1 (1:100) (Hup-1 N, Briar Patch Biosciences) at 4℃ overnight. After incubated with secondary antibody and counterstained with DAPI, pictures were taken with confocal microscopes (Olympics FV-1000).

#### PRM1 overexpression and knockdown

PcDNA3.3-PRM1 vectors were constructed by Liaoning Baihao Biotech Co., Ltd. (China). All siRNAs were purchased from Qiagen. Information was shown in Table S2. Transfections were performed using Lipofectamine 2000 (Thermo). Six hours later, medium was changed with FBS-reduced medium (1%FBS) with or without PRM1 protein/antibody.

#### Flow cytometry

Cells were incubated with PRM1 protein for 24 h and stained with PI or Annexin V- PE (C1052, C1062M, Beyotime, China). Cell cycle and apoptosis were analyzed by FACS (Beckman FC500, USA). All experiments were independently performed at least three times.

#### In vivo experiments

Balb/c nude mice were obtained from the Laboratory Animal Centre of Jilin University. SW480 cells (5 × 10^6^) were injected subcutaneously on the right side of the mouse’s back. PRM1 protein (2 μg/kg/d) and anti-PRM1 (44 μg/kg/d) were administrated intratumorally daily for 21 days. By the end of experiment, tumor growth curve and metastasis were monitored. All animal experiments were approved by the Use Committee for Animal Care of Jilin University.

#### Statistical analysis

Data was presented as mean ± standard error of the mean (SEM) and mean ± standard deviation (SD). Differences between two independent groups were tested with the student *t*-test or Mann–Whitney *U* test. Receiver operating characteristic (ROC) curves were constructed to assess areas under the curve (AUCs) with 95% CI. The correlation between serum PRM1 and clinicopathological characteristics was analyzed with Pearson`s χ^2^ test or Fisher exact test.

## Results

### Nine CRC-related CTA was identified by transcriptomic analysis

**Fig. 1:**
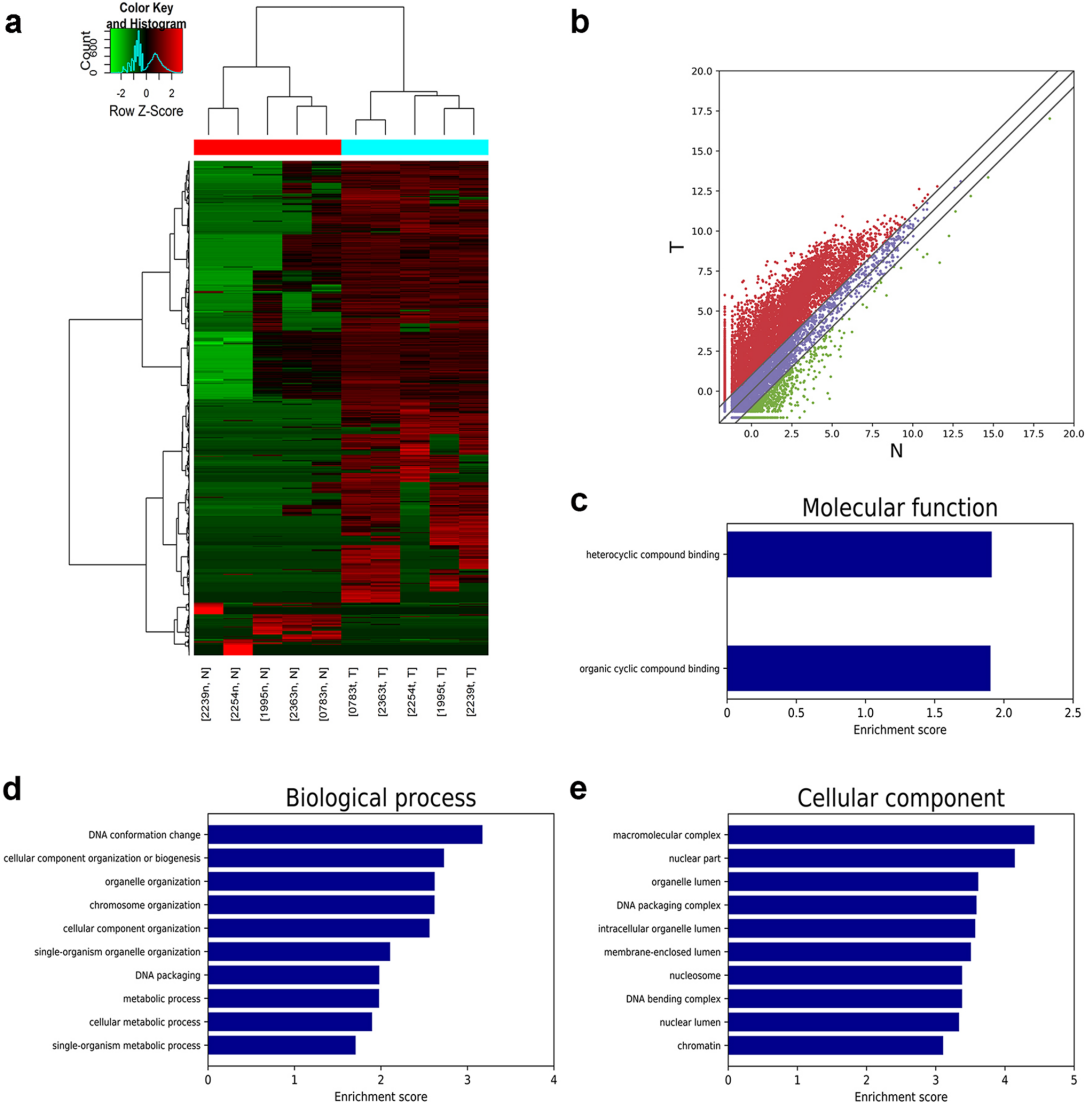
Transcriptomic analysis of 5 paired CRC tissues and PRM1 GO annotations (**a**) and (**b**) Heatmap and scatterplot of differentially expressed mRNAs. (**c-e**) GO annotations of PRM1

There were 1,160 mRNAs significantly upregulated, and 139 mRNAs downregulated in CRC tissues (*P* < 0.05) by transcriptomics analysis of five paired CRC tissues (Fig. 1a, b). Among them, we found that seven CTAs were upregulated and two were downregulated in CRC tissues (Table S3). Notably, POTEC and TDRD6 were first identified CTAs with increased expression in CRC tissues, and the cellular function will be studied in future research.

Next, we validated expression levels of these nine CTAs in CRC and other digestive tract cancer tissues aiming to screen a novel tumor marker with broad spectrum. The result showed that PRM1 was upregulated in CRC tissues, gastric cancer tissues, and esophagus cancer tissues (Fig. S1). The broad expression profile made PRM1 a very promising biomarker worthy of further research. Since firstly identified as a CTA in chronic lymphatic leukemia, the aberrant expression of PRM1 in CRC was reported previously by a small sampled study [17, 18]. However, no further exploration of diagnostic accuracy or biofunctions was ever attempted. So, we took PRM1 into further investigation and performed GO analysis, which revealed that PRM1 might be involved in several processes, including DNA conformation change, cellular component organization or biogenesis, and metabolic processes in CRC (Fig. 1c−e).

### PRM1 expression was upregulated in CRC tissues

**Fig. 2:**
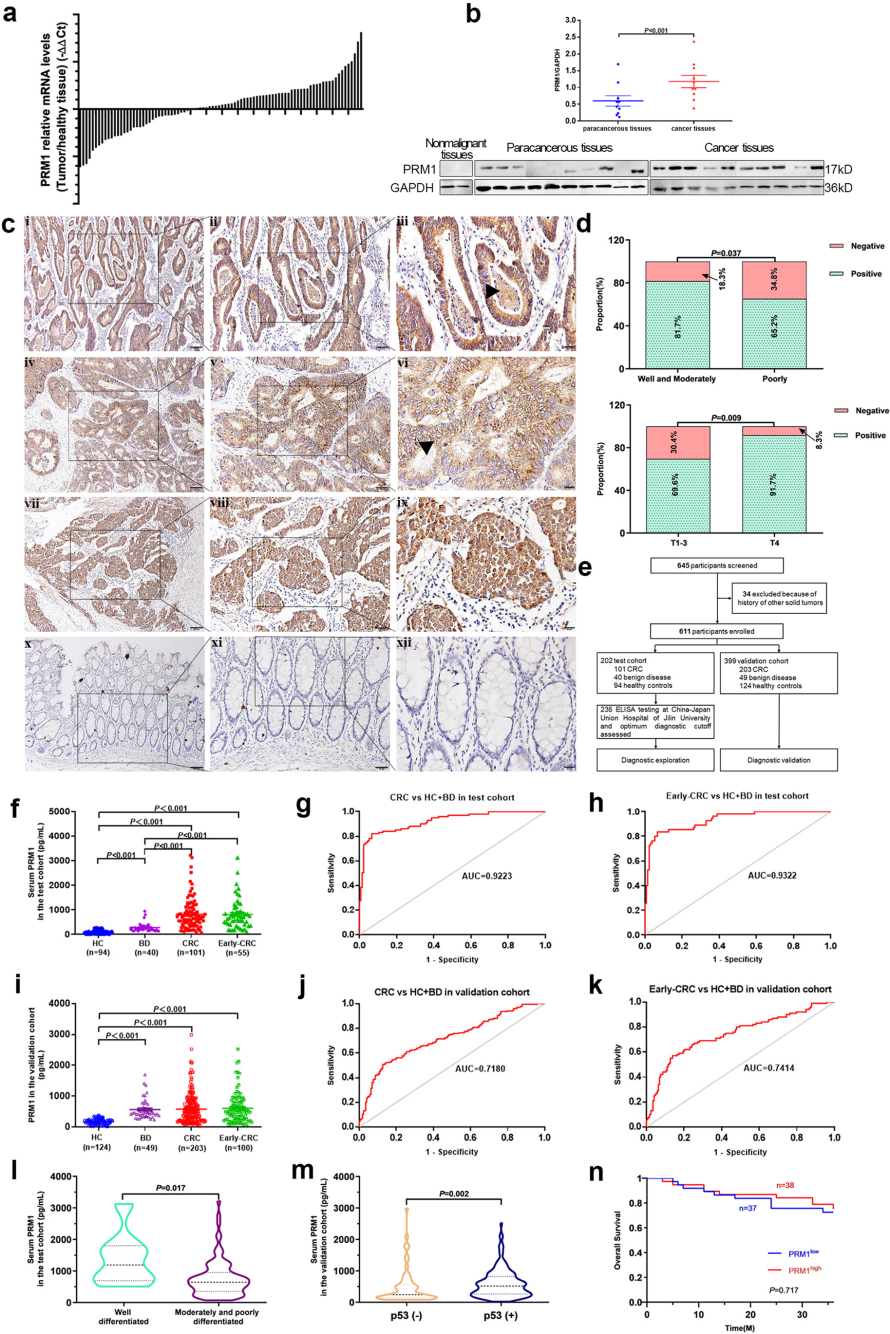
PRM1 is a potential marker for CRC diagnosis (**a**) PRM1 mRNA expression level in 90 CRC tumor tissues compared with paired adjacent normal tissues. (**b**) PRM1 protein expression in 10 paired colorectal cancer tissues and normal epithelial tissues from 2 nonmalignant patients. (**c**) Positive staining of PRM1 protein in CRC tumor tissues. Well-differentiated CRC tissues (i–iii), moderately-differentiated (iv–vi), poorly-differentiated CRC tissues (vii–ix) and normal colonic mucosal epithelium (x–xii); black arrow, positive staining of PRM1 in tumor cell cytosol and glandular lumens. PRM1 (yellow); Nucleus (blue). Scale bars: 100 μm in i, iv, vii, x; 50 μm in ii, v, viii, xi; 20 μm in iii, vi, ix, xii. (**d**) PRM1 protein expression were correlated with tumor differentiation level and T stage. (**e**) Study profile of the double-centered retrospective study. (**f**) Serum PRM1concentrations in CRC patients, BD patients, and HC from test cohort. (**g**) The sensitivity and specificity of serum PRM1 for CRC diagnosis in test cohort. **(h)** ROC curves of serum PRM1 for diagnosing all CRC and early-staged CRC in test cohort. (**i**) Serum PRM1 concentrations in CRC patients, BD patients, and HC from validation cohort. (**j**) The sensitivity and specificity of serum PRM1 in diagnosing CRC patients from validation cohort. (**k**) ROC curves of serum PRM1 for diagnosing all CRC and early-staged CRC in validation cohort. (**l**) Correlation of serum PRM1 with differentiation levels of CRC in test cohort. (**m**) Correlation of serum PRM1 with p53 status of CRC in validation cohort. (**n**) Kaplan–Meier curves for OS (overall survival) analysis of CRC patients in test cohort. BD, benign gastrointestinal disease; HC, healthy controls

To verify the upregulated expression of PRM1 in CRC, we collected a total of 90 paired CRC and adjacent normal tissues (defined as five cm from the tumor edge). Analyzed by qPCR, PRM1 mRNA expression was significantly elevated in 61.1% (55/90) of tumor tissues as compared with adjacent normal tissues (Fig. 2a). Next, we collected nonmalignant colonic epithelial tissues from two patients who underwent colectomy because of congenital megacolon and periappendiceal abscess, and performed western blot to compare PRM1 protein expression. Shown in Fig. 2b, PRM1 protein was not detected in colonic tissues from two benign disease patients, but was positive in all the ten cancer tissues. Remarkably, PRM1 protein levels were higher in CRC tissues than that in paired adjacent normal tissues. Additionally, by detection of three paired tissues from patients who were diagnosed simultaneously with CRC and colorectal adenoma, the expression level of PRM1 was very low in normal tissues, but it was upregulated in adenoma and the highest in cancer (Fig. S2). We then conducted immunohistochemistry (IHC) in 128 paraffin-embedded CRC tissues. Positive staining rate of PRM1 was 75.78% in CRC tissues (97/128), and negative staining was observed in paracancerous nonmalignant regions which indicated specific expression pattern of PRM1 (Fig. 2c, Fig. S3). Additionally, subcellular location of PRM1 protein was in CRC cytosol, and this was quite different from that in sperm nucleus.

Next, we analyzed the correlation of PRM1 expression level with CRC clinicopathological features. Significant relevance was observed between PRM1 protein staining rate and T stage (*P* = 0.009) and differentiation levels of CRC (*P* = 0.037), indicating that PRM1 might participate in tumorigenesis and development (Fig. 2d, Table 1). However, no relevance was observed between PRM1 mRNA levels and clinicopathological features (Table S4).

**Table 1:** Correlations between PRM1 protein expression and clinicopathologic characteristics of CRC detected by IHC

		PRM1 protein			
Variable	*n*	Negative (%)	Positive (%)	χ^2^	*P-*value
Age (y)				0.062	0.803
< 65	76	19(25.0%)	57(75.0%)		
≥ 65	52	12(23.1%)	40(76.9%)		
Gender				0.537	0.464
Male	67	18(26.9%)	49(73.1%)		
Female	61	13(21.3%)	48(78.7%)		
Differentiation level				4.366	0.037^*^
Well and moderately	82	15(18.3%)	67(81.7%)		
Poorly	46	16(34.8%)	30(65.2%)		
T-stage				6.887	0.009^*^
1–3	92	28(30.4%)	64(69.6%)		
4	36	3(8.3%)	33(91.7%)		
Lymph node metastasis				1.768	0.184
Negative	79	16(20.3%)	63(79.7%)		
Positive	49	15(30.6%)	34(69.4%)		
Tumor size (cm)				0.079	0.778
< 5	73	17(23.3%)	56(76.7%)		
≥ 5	51	13(25.5%)	38(74.5%)		
NA	4				
Vascular involvement				0.008	0.930
Negative	86	21(24.4%)	65(75.6%)		
Positive	38	9(23.7%)	29(76.3%)		
NA	4				
Perineurium invasion				0.059	0.809
Negative	93	22(23.7%)	71(76.3%)		
Positive	31	8(25.8%)	23(74.2%)		
NA	4				
Clinical stage				1.768	0.184
I–II	79	16(20.3%)	63(79.7%)		
III–IV	49	15(30.6%)	34(69.4%)		
EGFR				NO	0.305
Negative	113	26(23.0%)	87(77.0%)		
Positive	13	5(38.5%)	8(61.5%)		
NA	2				
P53				0.194	0.660
Negative	37	10(27.0%)	27(73.0%)		
Positive	90	21(23.3%)	69(76.7%)		
NA	1				
VEGF				NO	0.775
Negative	94	21(22.3%)	73(77.7%)		
Positive	20	5(25.0%)	15(75.0%)		
NA	14				
HER-2				NO	0.294
Negative	85	18(21.2%)	67(78.8%)		
Positive	14	1(7.1%)	13(92.9%)		
NA	29				

^*^Statistically significant

NA: not available

### Serum PRM1 was a promising diagnostic marker

Inspired by distinct cellular location in CRC cytosol and some glandular lumen, we hypothesized that PRM1 protein might be secreted by tumor cells to extracellular space and into patients’ circulation. Serum samples of 604 subjects were collected from two centers, and divided into two cohorts: 101 CRC patients, 40 benign gastrointestinal disease, and 94 healthy controls in test cohort; 203 CRC patients, 42 benign gastrointestinal disease, and 124 healthy controls in validation cohort (Fig. 2e).

After well matched for age and gender, we found that higher levels of serum PRM1 were observed in CRC patients than that in healthy controls (HCs) from both cohorts: 814.03 ± 625.37 vs 96.17 ± 88.53 pg/mL in test cohort (*P* < 0.001; Fig. 2f), and 577.5.35 ± 486.78 vs 179.79 ± 92.48 pg/mL in validation cohort (*P* < 0.001; Fig. 2i). Still, serum PRM1 was also influenced by some pathological conditions, such as infectious diseases (acute appendicitis, perianal abscess, et al.) and hyperplasia lesions (adenoma and polyps) (Fig. 2f and i).

Subsequently, we established ROC curves and calculated the diagnostic accuracy of serum PRM1 to distinguish CRC from HCs and BDs. AUCs were at 0.922 [95% CI, 0.887–0.957] and 0.727 [95% CI, 0.676–0.778] in test and validation cohort, respectively (Fig. 2g, j, and Table 2). Taken 301.61 pg/mL as optimum diagnostic cutoff in test cohort, we calculated sensitivity and specificity of serum PRM1 in both cohorts which revealed very promising diagnostic value (Table 2).

**Table 2:** Results for measurement of serum PRM1 in the diagnosis of CRC

Test	AUC (95%)	Sensitivity (%)	Specificity (%)	PPV (%)	NPV (%)	Positive LR	Negative LR
CRC vs HC + BD	0.962 (0.939–0.985)	84.2%	96.7%	96.6%	84.8%	25.809	0.164
Early-stage CRC vs HC + BD	0.971 (0.947–0.994)	85.5%	96.7%	94.0%	91.8%	26.206	0.150
Validation	AUC (95%)	Sensitivity (%)	Specificity (%)	PPV (%)	NPV (%)	Positive LR	Negative LR
CRC vs HC + BD	0.837 (0.782–0.892)	67.0%	85.5%	94.4%	41.2%	4.606	0.386
Early-stage CRC vs HC + BD	0.851 (0.791–0.910)	71.0%	85.5%	89.9%	61.8%	4.881	0.339

*AUC* area under curve, *PPV* positive predictive value, *NPV* negative predictive value, *LR* likelihood ratio, *CRC* colorectal cancer, *HC* healthy controls. *The diagnostic cutoff value was 271.94 pg/mL

### Serum PRM1 was more sensitive for early-staged CRC diagnosis

As improved CRC outcome dependents remarkably on early diagnosis, we attempted to explore whether serum PRM1 is a potential marker of early-staged CRC (T1-3N0M0). Shown in Fig. 2h and k, we observed a better performance of serum PRM1 in diagnosing early-staged CRC from BD and HCs in both cohorts: AUC = 0.932 in test cohort (95% CI [0.893–0.971]), AUC = 0.75 in the validation cohort (95% CI [0.686–0.813]). Particularly, diagnostic sensitivity was both elevated to 83.6% and 69%, respectively, implying serum PRM1 as a suitable marker for CRC early diagnosis (Table 2).

Since CEA, CA19-9, CA24-2, CA50, CA72-4 and AFP, are routinely used biomarkers for CRC diagnosis and recurrence monitoring, detection data of these markers was collected afterwards from test cohort. By establishment of ROC curves, CA50 showed highest AUC for CRC diagnosis at 0.76 which was still lower than that of PRM1 (Fig. S4). In future, combined detection of serum PRM1 and other biomarkers can be applied to improve diagnostic accuracy of CRC.

### Serum PRM1 was corelated with CRC differentiation level and p53 status

Similarly, we analyzed correlation of serum PRM1 with the clinicopathological features of CRC. Significant correlations were found between serum PRM1 and CRC differentiation level in test cohort (*P* = 0.017) (Fig. 2l), and p53 status in validation cohort (*P* = 0.002) (Fig. 2m). No association was found between serum PRM1 and age, gender, lymph node metastasis status, and other features of CRC (Table S5, S6; Fig. S5). Additionally, 3-year`s survival rate of CRC patients from test cohort was not affected by levels of serum PRM1 (Fig. 2n).

### CRC cell secreted PRM1 protein to extracellular space

**Fig. 3:**
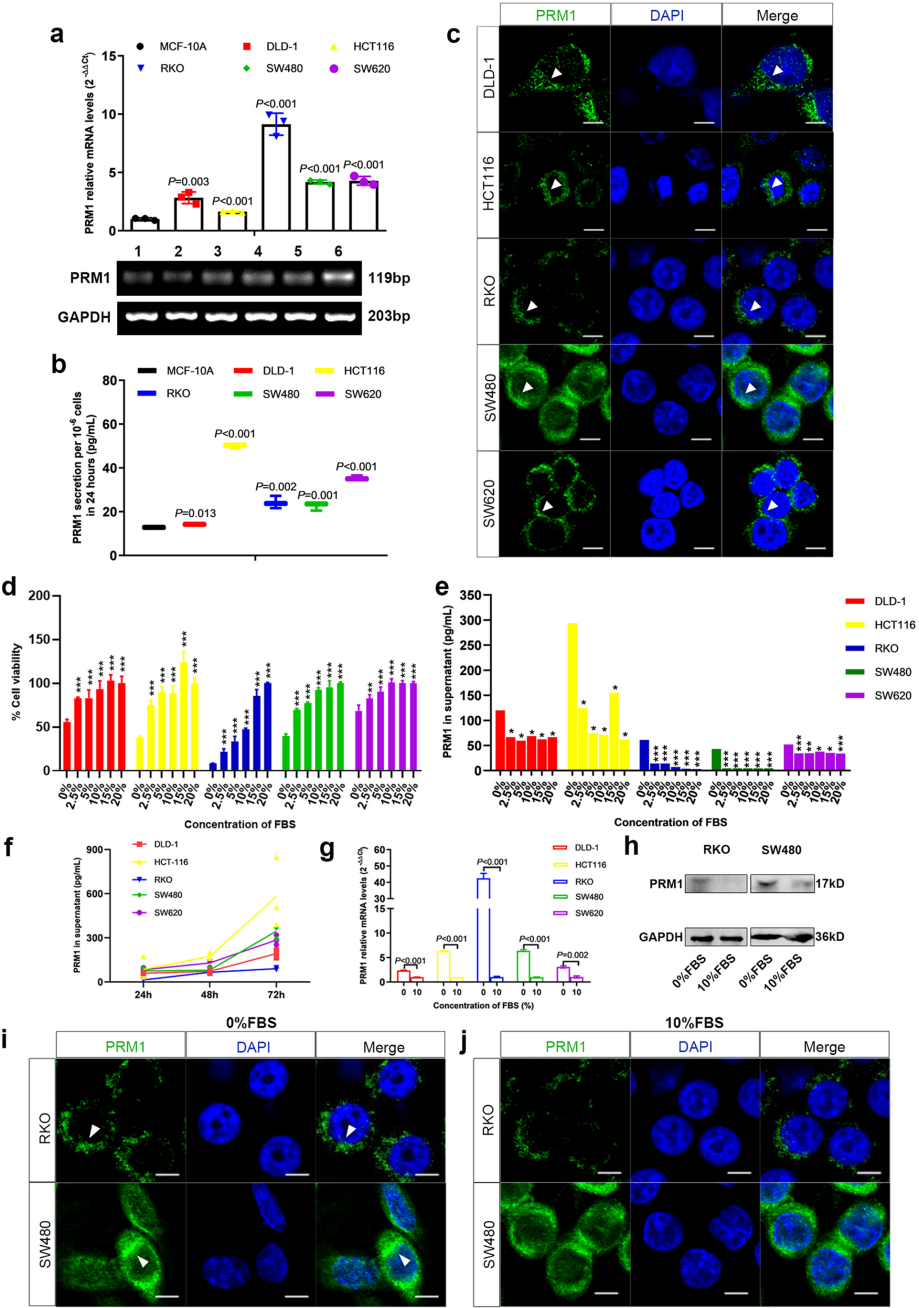
PRM1 expression and secretion were regulated by nutrition conditions (**a**) and (**b**) Elevated expression and secretion of PRM1 in CRC cells compared with MCF-10A. Lane 1 to 6 represent: MCF-10A, DLD-1, HCT116, RKO, SW480, SW620. (**c**) Subcellular location of PRM1 protein in CRC cytosol (white arrow). PRM1 (green); Nucleus (blue). Scale bars: 6 μm. (**d**) and (**e**) CRC cell viability and PRM1 secretion in 6 groups containing different concentrations of FBS; cells cultured in serum-free medium were used as control. (**f**) Persistent secretion of PRM1 by CRC cells cultured in serum-free medium. (**g**) and (**h**) Increased mRNA and protein expression of PRM1 in CRC cells when cultured in serum-free medium. (**i**) and (**j**) Comparison of PRM1 protein expression when cultured in complete medium or serum-free medium (white arrow). PRM1 (green); Nucleus (blue). Scale bars: 6 μm

To investigate biofunction of PRM1, we cultured five CRC cell lines (DLD-1, HCT116, RKO, SW480 and SW620). Compared with immortalized glandular epithelial cells MCF-10A, PRM1 expression and secretion were remarkably higher in CRC cells, showing that PRM1 as a secret protein of CRC cells (Fig. 3a, b). Besides, immunofluorescence (IF) also confirmed the subcellular localization of PRM1 protein in CRC cytosol which was in consistent with IHC results (Fig. 3c).

### PRM1 expression and secretion were upregulated under nutrient stress

It is well acknowledged that tumor cells always encounter hypoxia and nutritional deficiency during rapid growth, and initiates a serious of changes in gene expression to maintain cell viability. GO analysis has predicted that PRM1 might participate in metabolic processes in CRC. Therefore, we set up six groups in which cells were cultured in medium containing different concentrations of fetal bovine serum (FBS). The lower serum concentration represented the less nutrients. Although the proliferation rate of CRC cells declined sharply as FBS were reduced or absent (Fig. 3d), the expression and secretion of PRM1 were upregulated significantly, and persistently (Fig. 3e−h). Immunofluorescences also verified expression and location of PRM1 protein in RKO and SW480 cells when cultured under different nutrition conditions (Fig. 3i, j).

### Secreted PRM1 enhanced CRC proliferation under nutrition deficiency

**Fig. 4:**
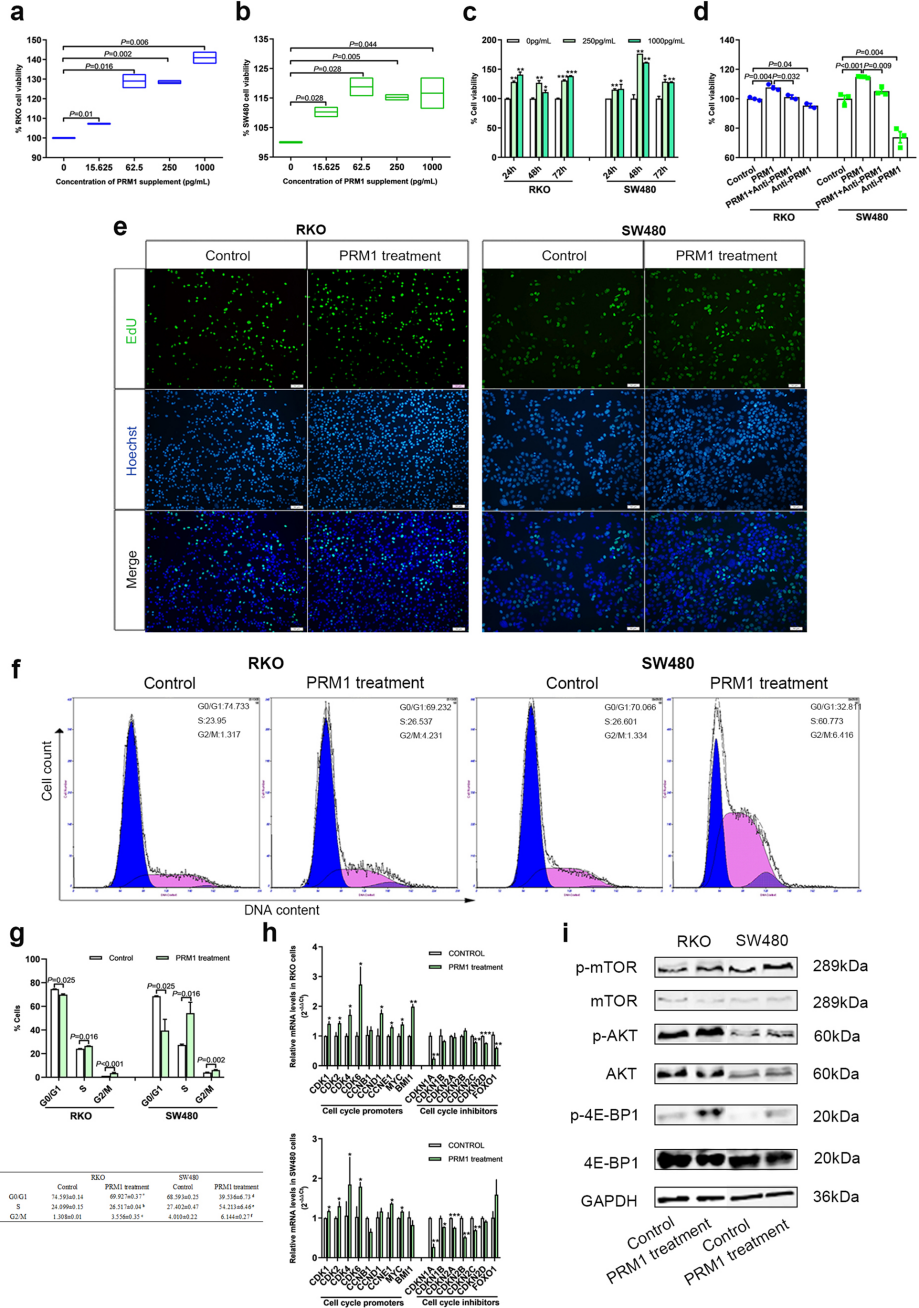
Extracellular PRM1 protein enhanced CRC cell proliferation under nutrition stress (**a-c**) PRM1 protein promoted CRC cell viability. Cells cultured in medium containing 1% FBS were used as control. (**d**) Antibody against PRM1 reversed the promoting effect of PRM1 protein on CRC cells. Cells cultured in medium containing 1% FBS were used as control. **(e)** PRM1 protein incubation elevated DNA replication level. Cells cultured in medium containing 1% FBS were used as control. EdU (green); Nucleus (blue). Scale bars: 50 μm. **(f)** PRM1 expression was associated with the enrichment of CELL_CYCLE gene set in CRC tissues. (**g**) and (**h**) PRM1 protein increased proportion of S phase in PRM1-treated groups. Cells cultured in medium containing 1% FBS were used as control. Data presented as Mean ± standard error of the mean (SEM). ^a^G1_RKO_ Control versus PRM1-treated, *P* = 0.025; ^b^S_RKO_ Control versus PRM1-treated, *P* < 0.001; ^c^G2_RKO_ Control versus PRM1-treated, *P* = 0 .016; ^d^G1_SW480_ Control versus PRM1-treated, *P* = 0.025; ^e^S_SW480_ Control versus PRM1-treated, *P* = 0.002; ^f^G2_SW480_ Control versus PRM1-treated, *P* = 0.026. (**i**) Expression levels of cell cycle promoters are upregulated, and levels of inhibitors are downregulated in PRM1-treated groups. Cells cultured in medium containing 1% FBS were used as control. (**j**) Activation of PI3K/AKT/mTOR pathway in CRC cells after PRM1 protein treatment. Cells cultured in medium containing 1% FBS were used as control

To learn more about the increased secretion of PRM1 under nutrient stress conditions, RKO and SW480 cells were cultured in serum-deficient environment (medium containing 1%FBS), and PRM1 protein was added to the culture system. By CCK8, we found remarkably increased cell viability in PRM1 protein treated group at all four concentrations (Fig. 4a−c). Meanwhile, antibody against PRM1 neutralized secreted PRM1 protein to inhibit CRC growth effectively (Fig. 4d, Figure S6).

Subsequently, we conducted EdU assays and found that DNA replication level was enhanced in PRM1 protein incubation group when cells were cultured in medium containing 1% FBS (Fig. 4e). To further clarify the mechanism of secreted PRM1 on cell proliferation, we applied flow cytometry and found significant progression of G1/S phase transition in PRM1 protein incubation group (Fig. 4f, g). Consistently, expression of cell cycles promoters was also upregulated, and that of cell cycle inhibitors was downregulated in PRM1 incubated group as compared with control group (Fig. 4h).

In terms of signal transduction, PI3K/AKT/mTOR pathway is a well-known pathway which receives signals of growth factor from extracellular space and regulate cell proliferation, apoptosis, cell cycle and other cellular events [19, 20]. We then conducted western blot and found increased levels of p-AKT [Ser473], p-mTOR [Ser2448], and p-4E-BP1 [Thr37/46] in PRM1 protein incubated group. The data indicated that secreted PRM1 functions as an activator of PI3K/AKT/mTOR pathway under nutrition deficient context (Fig. 4i).

On the other hand, we also asked whether PRM1 had effect on CRC apoptosis and metastasis when cells were cultured under nutrient stress. Analyzed by flow cytometry, wound healing, and transwell assays, data showed that PRM1 had no influence on metastasis abilities or cell apoptosis (Figure S7, S8).

### Proliferation rate of CRC cells was enhanced after PRM1 overexpression

**Fig. 5:**
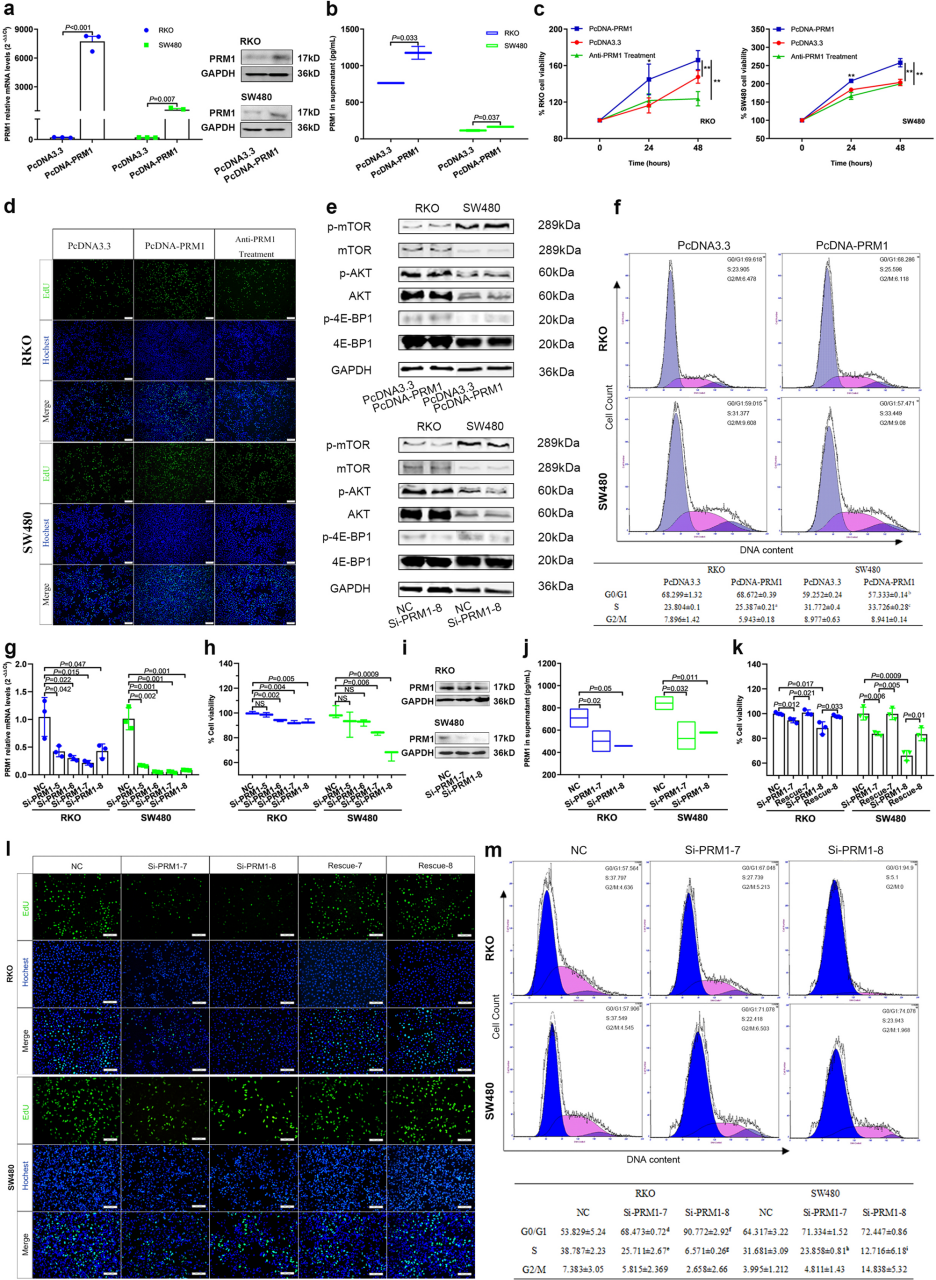
Manipulation of PRM1 gene expression influenced CRC cell proliferation under nutrient deficiency conditions (**a**) and (**b**) Increased expression and secretion of PRM1 after PcDNA-PRM1 transfection. (**c**) Enhanced cell viability after PRM1 overexpression which was antagonized by anti-PRM1 incubation. (**d**) Increased levels of DNA replication after PRM1 overexpression. EdU (green); Nucleus (blue). Scale bars: 100 μm. (**e**) Activation levels of PI3K/AKT/mTOR pathway after PRM1 knockdown and overexpression. (**f**) Progression of G1/S phase transition of CRC cells after PRM1 overexpression. Data presented as Mean ± SEM. ^a^S_RKO_ PcDNA3.3 versus PcDNA-PRM1, *P* = 0.021; ^b^G1_SW480_ PcDNA3.3 versus PcDNA-PRM1, *P* = 0.02; ^c^S_SW480_ PcDNA3.3 versus PcDNA-PRM1, *P* = 0.028. (**g**) and (**h**) Changes of PRM1 expression and cell viability after transfection of si-RNAs. (**i**) and (**j**) Decreased expression and secretion of PRM1 after knockdown; cells transfected with negative control were used as control. (**k**) and (**l**) Inhibited viability and DNA replication of CRC cells after PRM1 knockdown, which was rescued by PRM1 protein supplement. (**m**) Cell cycle arrested at G1/S phase after PRM1 knockdown. Data presented as Mean ± SEM. ^**d**^G1_RKO_ NC versus Si-PRM1-7, *P* = 0.025; ^**e**^S_RKO_ NC versus Si-PRM1-7, *P* = 0.01; ^**f**^G1_RKO_ NC versus Si-PRM1-8, *P* = 0.014; ^**g**^S_RKO_ NC versus Si-PRM1-8, *P* = 0.002; ^**h**^S_SW480_ NC versus Si-PRM1-7, *P* = 0.034; ^**i**^S_SW480_ NC versus Si-PRM1-8, *P* = 0.026

Subsequently, we sought to testify the cellular effect of secreted PRM1 protein on CRC cells by manipulating endogenous expression of PRM1. PcDNA-PRM1 was transfected into RKO and SW480 cells, and the medium was changed with serum-reduced medium (1% FBS) at six hours after transfection. After confirmation of increased PRM1 expression and secretion after transfection (Fig. 5a, b), we observed enhanced proliferation rate of CRC cells as compared with vector control group (Fig. 5d, d). Additionally, anti-PRM1 was used as an antagonist and added to culture medium after PRM1 overexpression, then inhibited cell proliferation and reduced DNA replication were observed (Fig. 5c, d). Western blot and flow cytometry in turn revealed activation of PI3K/AKT/mTOR pathway and progression of G1/S phase transition after PRM1 overexpression when cells were cultured in serum deficient medium (Fig. 5e, f).

#### Secreted PRM1 serves as therapeutic target of CRC metabolism rewiring

Conversely, siRNAs targeting PRM1 sequence were ordered from Qiagen. Co., and transfection was conducted in the same way as PRM1 overexpression. Six hours after transfection, culture medium was changed with medium containing 1%FBS. PRM1 expression were downregulated after si-PRM1s transfection (Fig. 5g). PRM1 secretion and cell proliferation rate were all declined obviously in si-PRM1-7 and si-PRM1-8 transfection groups (Fig. 5h−j). Besides, we conducted rescue experiments by adding PRM1 protein to the culture medium after transfection. Shown in Fig. 5k and l, the inhibited cell proliferation and DNA replication level were all rescued by PRM1 protein supplementation. Meanwhile, activation of PI3K/AKT/mTOR pathway and G1/S phase transition were all impaired by PRM1 knockdown when cells were cultured in serum deficient environment (Fig. 5e, m).

#### Secreted PRM1 protein facilitates CRC Growth in vivo

**Fig. 6:**
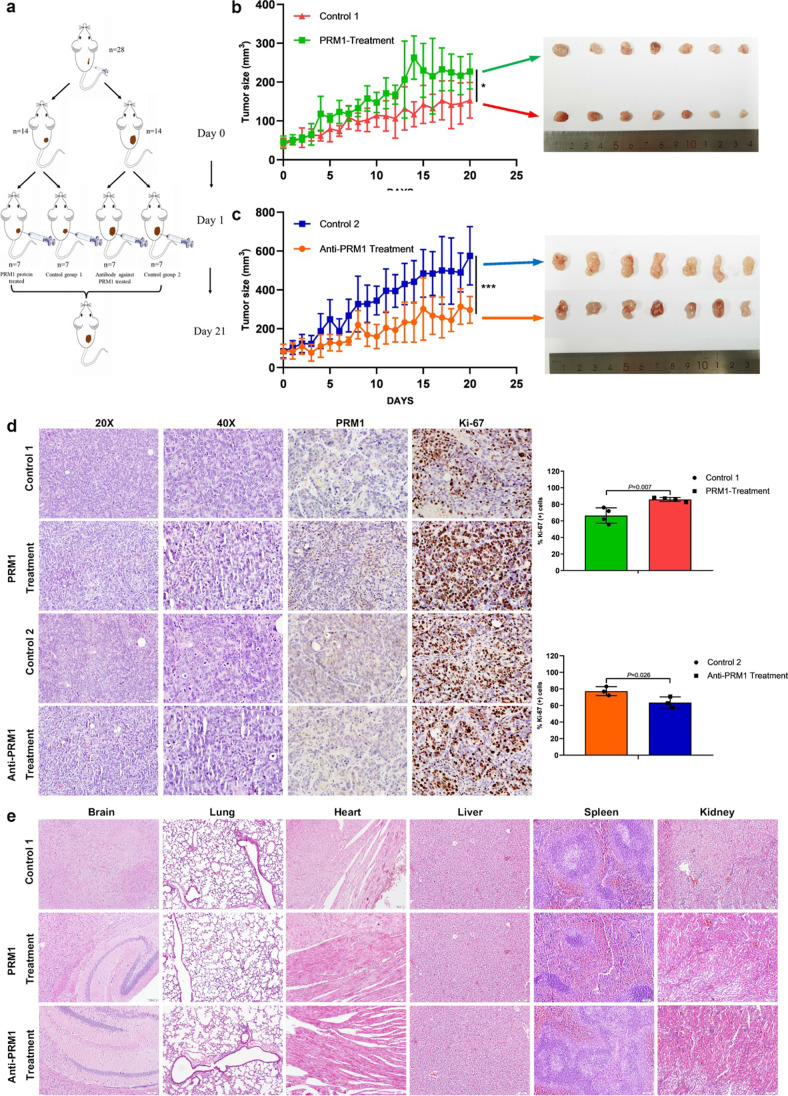
PRM1 protein and anti-PRM1 had impact on CRC growth in vivo (**a**) Schematic diagram of animal experiment. (**b**) Growth curve of mice in PRM1 protein injection group and control group 1 (left). Gross observation of tumors in the two groups (right). (**c**) Growth curve of mice in anti-PRM1 injection group and control group 2 (left). Gross observation of tumors in the two groups (right). (**d**) H&E, PRM1, and Ki-67 staining of tumor xenograft tissues, and the column chart of Ki-67 staining rate in PRM1 protein-treated group and antibody-treated group. Scale bars, 50 μm and 20 μm. (**e**) H&E staining of organs. No signs of metastasis or tissue toxicity was observed in each group. Scale bars, 50 μm

Lastly, we sought to validate the effect of secreted PRM1 on CRC growth in vivo. CRC xenograft was established in Balb/c nude mice by subcutaneously injection of SW480 cells. After tumor formation, mice were grouped into two cohorts and received corresponding treatments as follows: mice harboring smaller tumors (mean volume 44.02mm^3^, *n* = 14) were injected intratumorally with PRM1 protein or PBS (control group 1), and those harboring larger tumors (mean volume 84.05mm^3^, *n* = 14) were treated with antibody against PRM1 or PBS (control group 2) (Fig. 6a). After treated for 20 days, tumors stimulated by PRM1 protein grew remarkably faster and were larger than that of control group 1, and tumors treated with antibody grew slower and were smaller than corresponding control group 2 (Fig. 6b, c). Additionally, tumors stimulated by PRM1 protein exhibited higher staining rate of Ki67(*P* = 0.007), and lower staining rate of Ki67 was observed in tumors treated with anti-PRM1(*P* = 0.026) (Fig. 6d). Besides, HE staining of mice organs also revealed no signs of tumor metastasis in all four groups. No signs of tissue damage or infiltration of immune cells was detected in mice received antibody treatment, implying the biosafety of antibody treatment (Fig. 6e).

On the other hand, we also explored the function of anti-PRM1 during tumorigenesis. Balb/c nude mice were inoculated with mixture of SW480 cells and anti-PRM1. Tumor formation rate was slightly lower in antibody treated group (6/15) as compared with control group (5/10), and tumor growth was significantly inhibited by antibody treatment, which suggested that antibody against PRM1 does not only inhibit CRC growth, but also have a certain impact on tumorigenesis (Fig. S9).

## Discussions

Although electronic coloscopy is currently believed to be reference method for CRC screening and diagnosis, the application is still limited for the invasive nature, bowel preparation, and relative high cost [21]. Serological detection is a promising candidate method, and it is critical to explore more sensitive markers to improve detection accuracy. So far, over 200 CTAs have been identified in many malignancies, and are considered as optimal diagnostic and therapeutic targets [22]. Colorectal cancer, however, is characterized with low expression of CTAs, and fewer CTAs exhibit promising diagnostic potentials. In the previous studies, MAGE family, SPAG9, AKAP4, PRM1, et al., were all expressed in CRC, with the expression frequency ranging from 44 to 70%, however, the sample sizes used for detection were relatively small, and the diagnostic value as well as cellular functions were not further explored.

In this report, CRC-related CTA profile was broadened through transcriptome sequencing, with TDRD6 and POTEC as firstly identified CRC-related CTA. Aiming to explore novel biomarker with broad expression profile in digestive tract cancers, we revealed PRM1 upregulated in CRC, gastric cancer, and esophagus cancer. By further analysis of 218 clinical samples, we reported PRM1 mRNA and protein expression frequencies at 61.1% (55/90) and 75.78% (97/128), respectively, which were much higher than other CTAs in literature. Especially, no PRM1 protein was stained in nonmalignant colorectal tissues. On the other hand, we uncovered the promising diagnostic accuracy of serum PRM1 for CRC diagnosis. Similar to commonly used tumor markers, levels of serum PRM1 was also influenced by some pathological conditions, including inflammatory and benign proliferation lesions. As adenoma and inflammatory bowel disease (ulcerative colitis and Crohn`s disease) are well-accepted risk factors for CRC development [23, 24], we found increasing trend of PRM1 expression in colorectal adenoma and cancer tissues, and it is worthy to further investigate whether PRM1 can be used as an alarm marker for CRC. Besides, we observed higher sensitivity for serum PRM1 to diagnose early-staged CRC (T1-3N0M0), and larger sampled clinical studies as well as well-designed in vivo assays are in want to clarify the tendency of serum PRM1 during tumorigenesis. In clinical practice, joint detection of several markers is essential to improve diagnostic accuracy. Due to the co-expression pattern of many CTAs in tumors [25], it is promising to develop PRM1-based CTA-panel for CRC early diagnosis to improve efficacy.

During the rapid growth and progression of tumor, metabolic stress is always encountered including nutrition defects, hypoxia, and acidic environment. Cancer cells must take alterations of metabolic state to overcome the metabolic challenges, and metabolic reprogramming is currently considered as a hallmark of tumor growth and development [26]. As nutrition composition changes a lot in tumor microenvironment, gene expression reprogramming happens in cancer cells to facilitate survival and growth under harsh environment. Breast cancer cells upregulated LLGL2 expression to increase uptake of leucine under nutrient stress which is an essential amino acid for cell metabolism [27]. Lipid metabolism is also an important aspect of tumor metabolic rewiring. PCYT2 was recently reported to be downregulated in cancer cells under glutamine starvation which led to accumulation of phosphoethanolamine (PEtn), and PEtn in turn enhanced the tolerance of cancer cells to starvation [28]. Besides, recent researches also revealed that several CTAs also play regulatory roles in tumor metabolism [29–31]. Importantly, SEMG1 and SEMG2 were responsible for protein level and activity of two main glycolysis enzymes to increase the membrane mitochondrial potential (MMP) and ROS production. Noteworthy, PRM1 was reported to be interacted with SEMG1 in chronic leukemia, which implied the functional relevance between PRM1 and SEMG1 [18]. In this study, PRM1 expression and secretion were upregulated remarkably when CRC cells were cultured in serum-free medium, suggesting that PRM1 is involved in CRC metabolism rewiring. In the following part, biofunctions of PRM1 was identified by PRM1 protein or anti-PRM1supplementation which had significant influence on cell proliferation and cell cycles. In terms of mechanism, many extracellular signals including growth factors regulate cellular processes via PI3K/AKT/mTOR pathway [32–37]. Herein, we observed increased activation of PI3K/AKT/mTOR pathway after PRM1 overexpression and protein supplementation. In that case, it was first proposed that secreted PRM1 participate in CRC metabolism reprogramming to facilitate cell proliferation under harsh environment.

In current reports, researchers have summarized the biofunctions of CTAs into three aspects: transcriptional regulation, mitotic fidelity, and protein degradation [6]. ZNF165 (C2H2 zinc finger transcription factor, also known as CT53) regulates the expression of genes in TGFβ pathway to promote tumorigenesis [38, 39]; TEX14, CASC5, TTK, and NUF2 all participate in the kinetochore assembly process of tumor cells [40, 41]; MAGE-A3/6 interacts with TRIM28 to regulate the proteasome-dependent degradation of tumor suppressors [30, 42]. PRM1, however, acts as a DNA binding protein in spermatogenesis, and its aberrant location into somatic cell nucleus will lead to chromatin condensation and impair proliferation of Hela and *E.coli* [43, 44]. It excludes the possibility that PRM1 could participate directly in transcriptional regulation or mitotic fidelity maintenance of CRC cells, and it also explains why PRM1 is only expressed in the cytosol of CRC cells. Functional research carried out in this work revealed that PRM1 is involved in tumor growth regulation, especially under nutrient limitation. Secreted by CRC cells, PRM1 might be considered as a growth factor to activate PI3K/AKT/mTOR pathway stimulating tumor growth. As changes of cell proliferation observed after PRM1 overexpression or knockdown were all inhibited and rescued by addition of antibody and PRM1 protein to the supernatant, it suggested that PRM1 regulates CRC cell growth in an autocrine and paracrine manner. Interaction between PRM1 and membrane receptor plays essential role in regulation of CRC growth, which remains to be further explored.

Owing to the specific expression pattern and immunogenicity, CTAs are attractive targets of cancer immunotherapy, including T-cell receptors (TCRs), CAR T cell, antibody-based therapy, and cancer vaccines [1]. Till now, numerous clinical trials have been carried out to validate the efficacy of CTA-targeted immunotherapy, and clinical translation remains the major issue which is hindered by low immunogenicity and complex tumor microenvironment [45–47]. Although most clinical trials of CTA-based immunotherapy in the treatment of melanoma and lung cancer, studies on the treatment of CRC by targeting CTA are very limited. Immunotherapy targeting MAGE has achieved ideal results in treatment of metastatic CRC, and entered phase II clinical trial [48, 49]; the polypeptide vaccine based on HSP105 (heat shock protein family h member 1) induced strong immune response and was used to treat advanced CRC [50]. In this study, we found PRM1 with specific expression pattern in CRC, which laid foundations to further design PRM1-based immunotherapy. Cancer vaccine composed of PRM1 epitope may facilitate tumor control by induction of specific immune response, since we have demonstrated that anti-PRM1 can be used as an antagonist of secreted PRM1 to inhibit CRC tumorigenesis and cell proliferation.

However, there are still some limitations in this study. Due to time and economic factors, we did not further validate the diagnostic potential of PRM1 in a large sampled prospective clinical study, and administration of antibody in vivo needs more optimization. In future research, multicentered prospective clinical studies are needed to justify the diagnostic value of PRM1, as well as the specific molecular mechanism of PRM1 in cell metabolism rewiring under harsh conditions. Intramolecular interaction of PRM1 and membrane receptors are warranted to clarify underlying mechanism of PRM1 as a growth factor during development of CRC. Efforts will also be taken to develop PRM1-based tumor immunotherapy strategies, either in forms of cancer vaccine or antibody-based treatment.

In general, CTA expression profile in CRC was expanded through RNA-sequencing, and the impressive diagnostic value of PRM1 was also demonstrated for early-staged CRC. Induction of PRM1 expression was proved to function in a growth factor-like manner to support CRC growth during metabolism rewiring under nutrition deficient conditions. PRM1 also represents a new vulnerability of CRC cells with the potential to be targeted.

## Supplementary Information

Below is the link to the electronic supplementary material.

Supplementary file1 (DOCX 1234 KB)

## Data Availability

The RNA-seq data was deposited in the National Center for Biotechnology Information (NCBI) Gene Expression Omnibus (GEO) under the number of GSE141746).

## Abbreviations

*AUC*: Area under the curve
*BMI1*: The polycomb group protein B lymphoma Mo-MLV insertion region 1 homolog
*CA19-9*: Carbohydrate antigens 19–9
*CEA*: Carcinoembryonic antigens
*CLL*: Chronic lymphocytic leukemia
*COX6B2*: Cytochrome c oxidase subunit 6B2
*CRC*: Colorectal cancer
*CTA*: Cancer-testis antigen
*DAVID*: Database for Annotation, Visualization, and Integrated Discovery
*EGFR*: Epidermal growth factor receptor
*FOXO1*: Forkhead box O1
*GEO*: Gene expression omnibus
*GSEA*: Gene set enrichment analysis
*GO*: Gene Ontology
*HC*: Healthy controls
*HER-2*: Human epidermal growth factor receptor-2
*IDPs*: Intrinsically disordered proteins
*KEGG*: Kyoto Encyclopedia of Genes and Genomes
*Ki67*: Antigen KI-67
*MAGE*: Melanoma associated antigen
*NY-ESO-1*: New York esophageal squamous cell carcinoma 1
*PCNA*: Proliferating cell nuclear antigen
*POTEC*: POTE ankyrin domain family member C
*PRM1*: Protamine 1
*PRM2*: Protamine 2
*ROC*: Receiver operating characteristic
*SPAG9*: Sperm-associated antigen 9
*TDRD6*: Tudor domain containing 6
*VEGF*: Vascular endothelial growth factor
