# Supplementary material for: Protamine 1 as a secreted colorectal cancer-specific antigen facilitating G1/S phase transition under nutrient stress conditions
Source: Cell Oncol (Dordr). 2023 Jan 3;46(2):357–73. doi: 10.1007/s13402-022-00754-w (PMC10060357; doi:10.1007/s13402-022-00754-w)
Supplement: Supplementary file 1 — Supplementary file1 (DOCX 1234 KB) [file 13402_2022_754_MOESM1_ESM.docx]

**Supplemental Figures:**


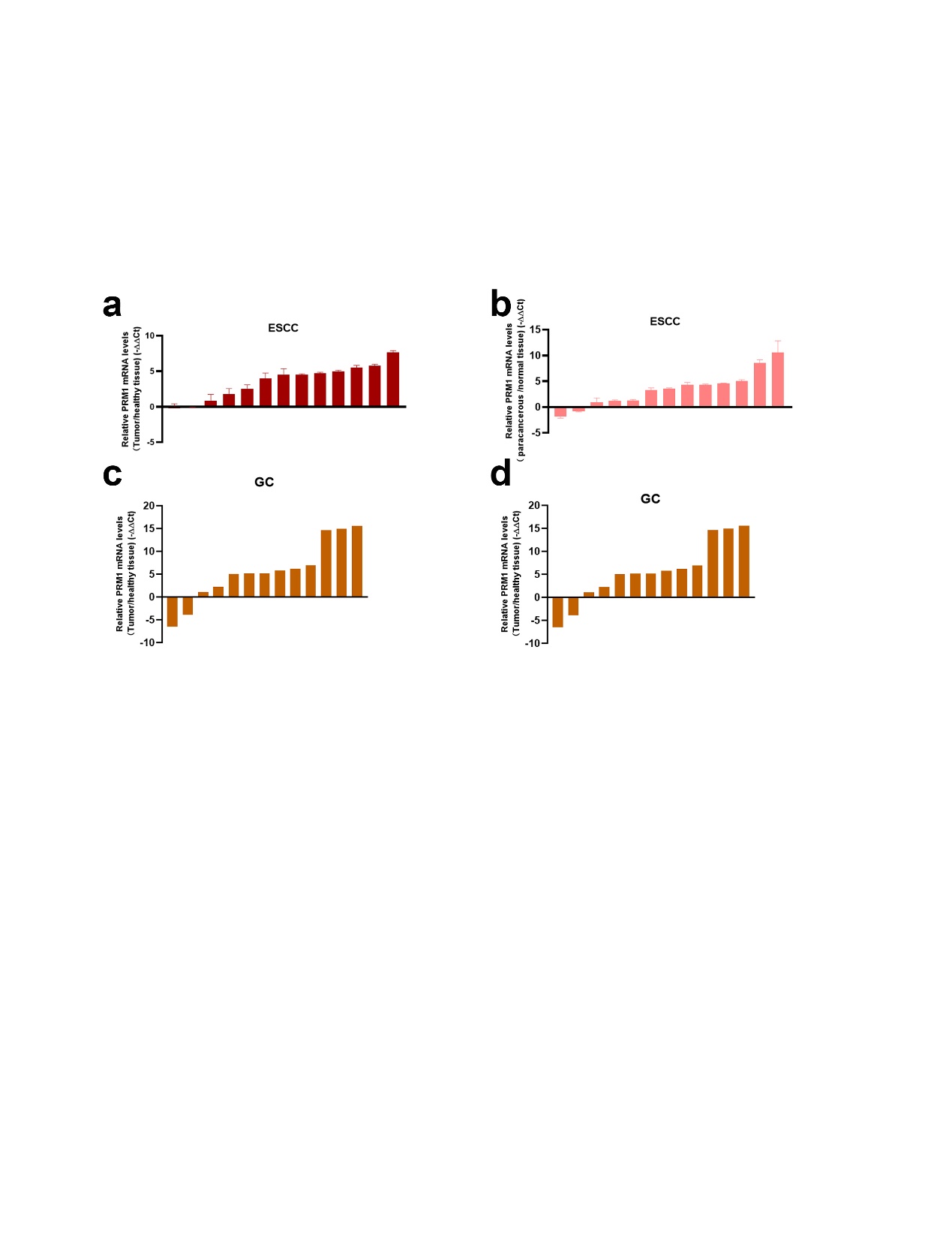


Fig. S1 PRM1 mRNA expression was upregulated in cancer tissues and paracancerous tissues from esophageal squamous cell carcinoma patients (a, b), and gastric adenocarcinoma patients (c, d).


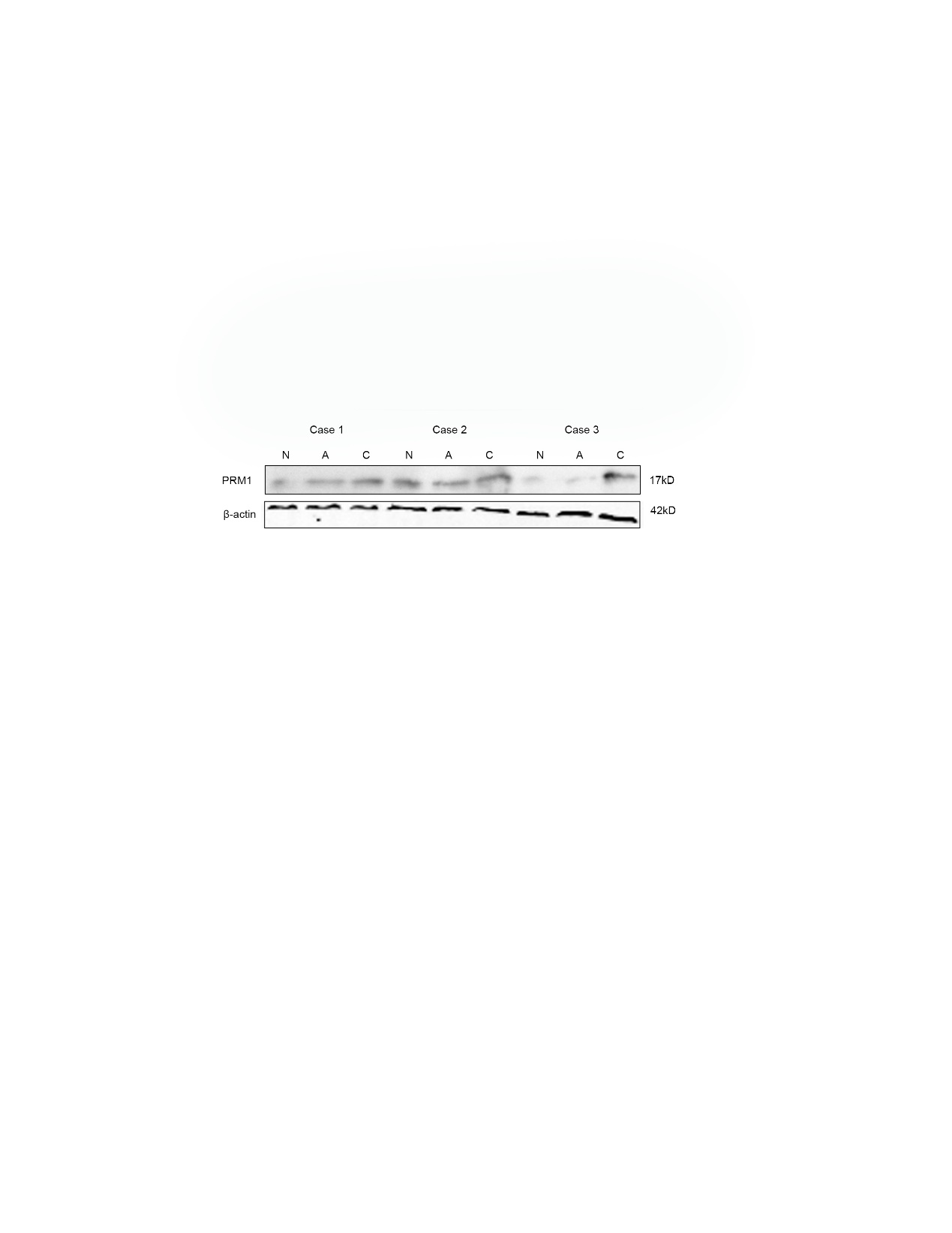


Fig. S2 PRM1 protein expression in three paired CRC tissues. Patients were all diagnosed with CRC and single or multiple adenomas simultaneously. N represents normal tissue; A represents adenoma; C represents cancer tissue.


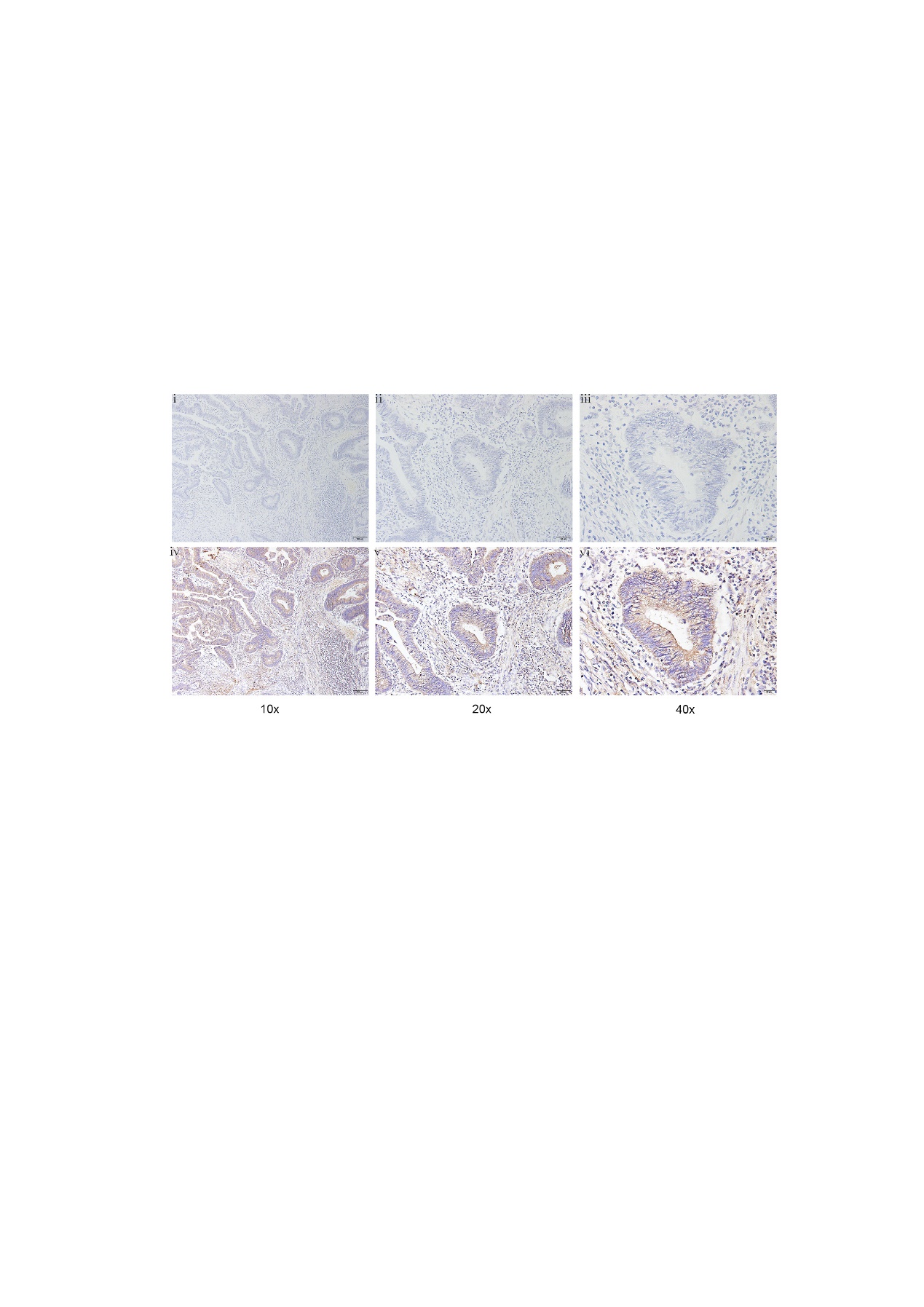


Fig. S3 Negative control for IHC staining of PRM1 in CRC tissues (ⅰ–ⅲ) incubation without antibody against PRM1, (ⅳ–ⅵ) incubation with antibody against PRM1. PRM1 (yellow); Nucleus (blue). Scale bars: 100μm in ⅰ, ⅳ; 50μm in ⅱ, ⅴ; 20μm in ⅲ, ⅵ.


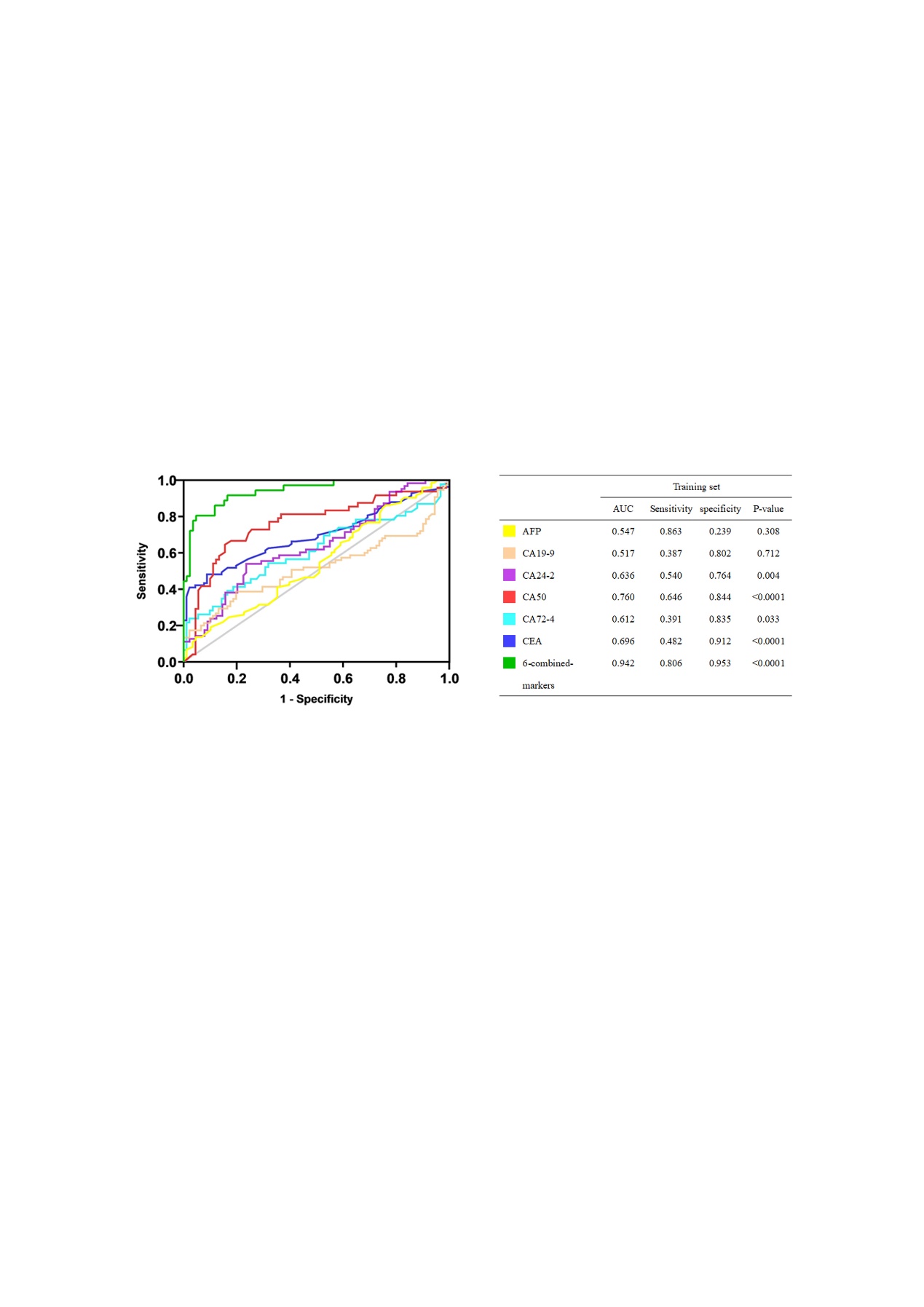


Fig. S2 ROCs of AFP, CA19-9, CA24-2, CA50, CA72-4, CEA, either used individually or in combination to diagnose CRC from healthy controls


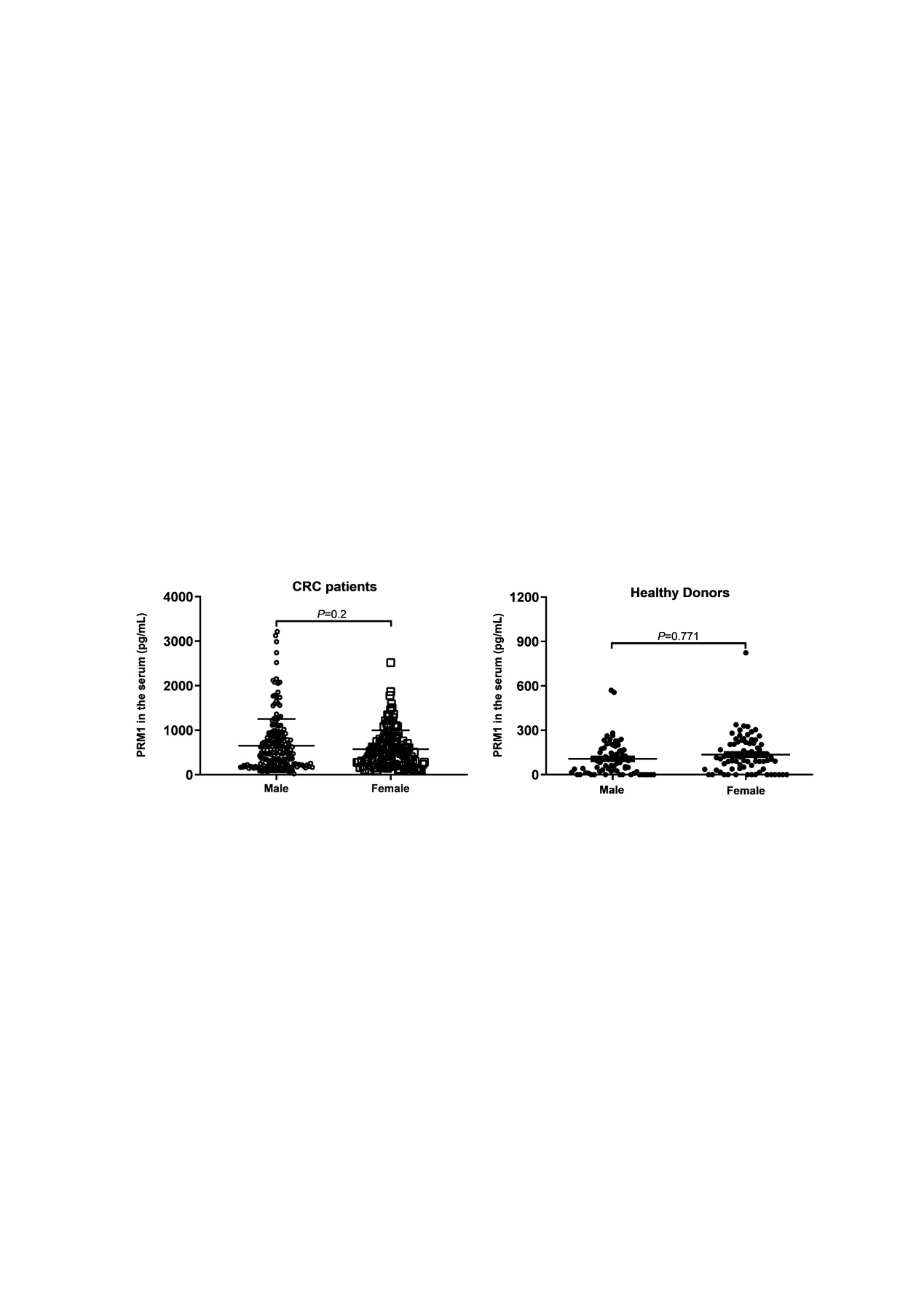


Fig. S5 Concentrations of serum PRM1 showed no difference between males and females


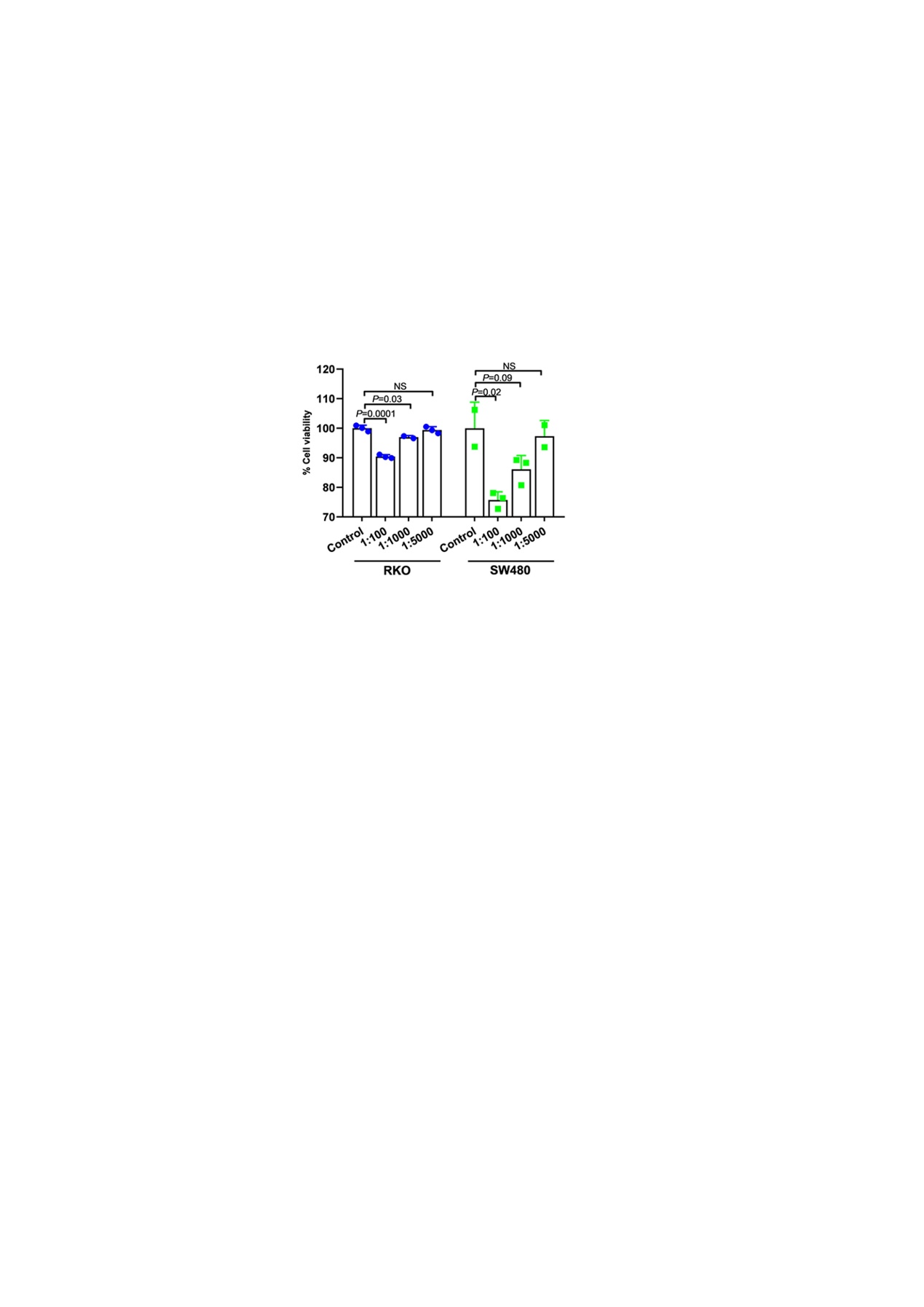


Fig. S6 Antibodies against PRM1 inhibited CRC cell proliferation in a concentration-dependent manner Cells cultured in RPMI1640 medium containing 1% FBS are used as control.


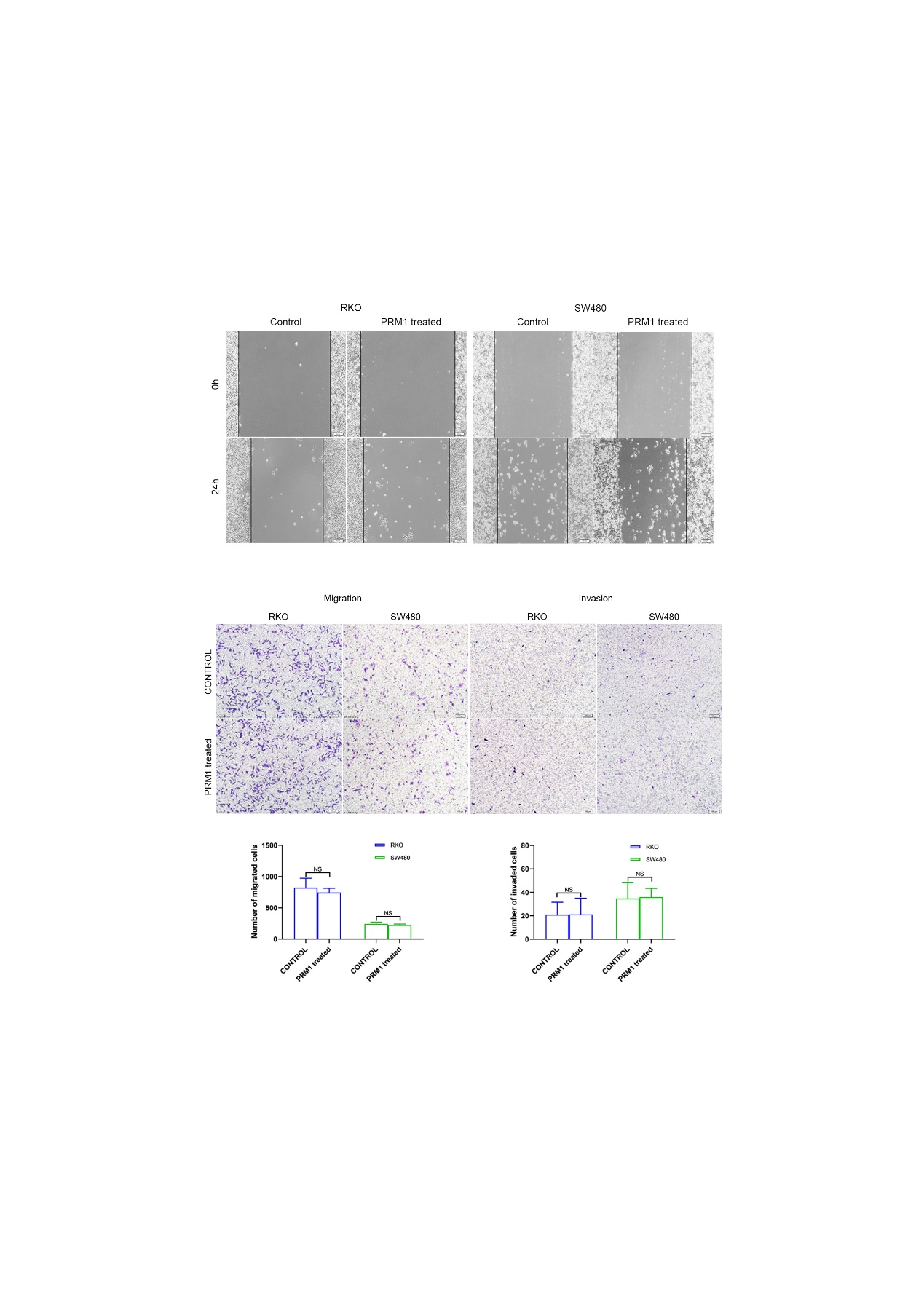


Fig. S7 Wound healing and transwell assays revealed no differences on CRC metastasis after PRM1 treatment Cells cultured in RPMI 1640 medium containing 1% FBS are used as control. Scale bars: 100μm.


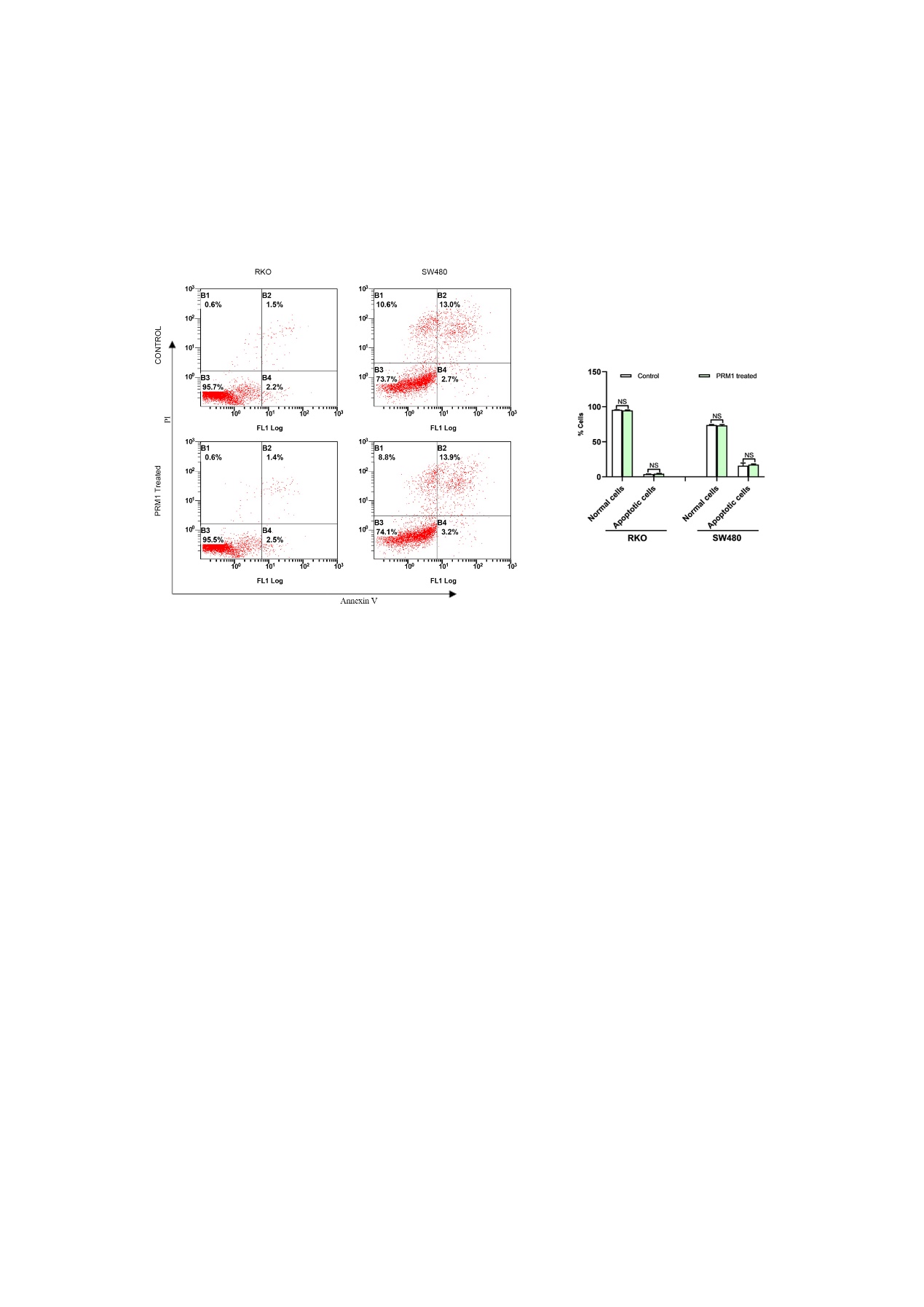


Fig. S8 PRM1 protein incubation did not induce CRC cell apoptosis Data presented as Mean ± standard error of the mean (SEM).


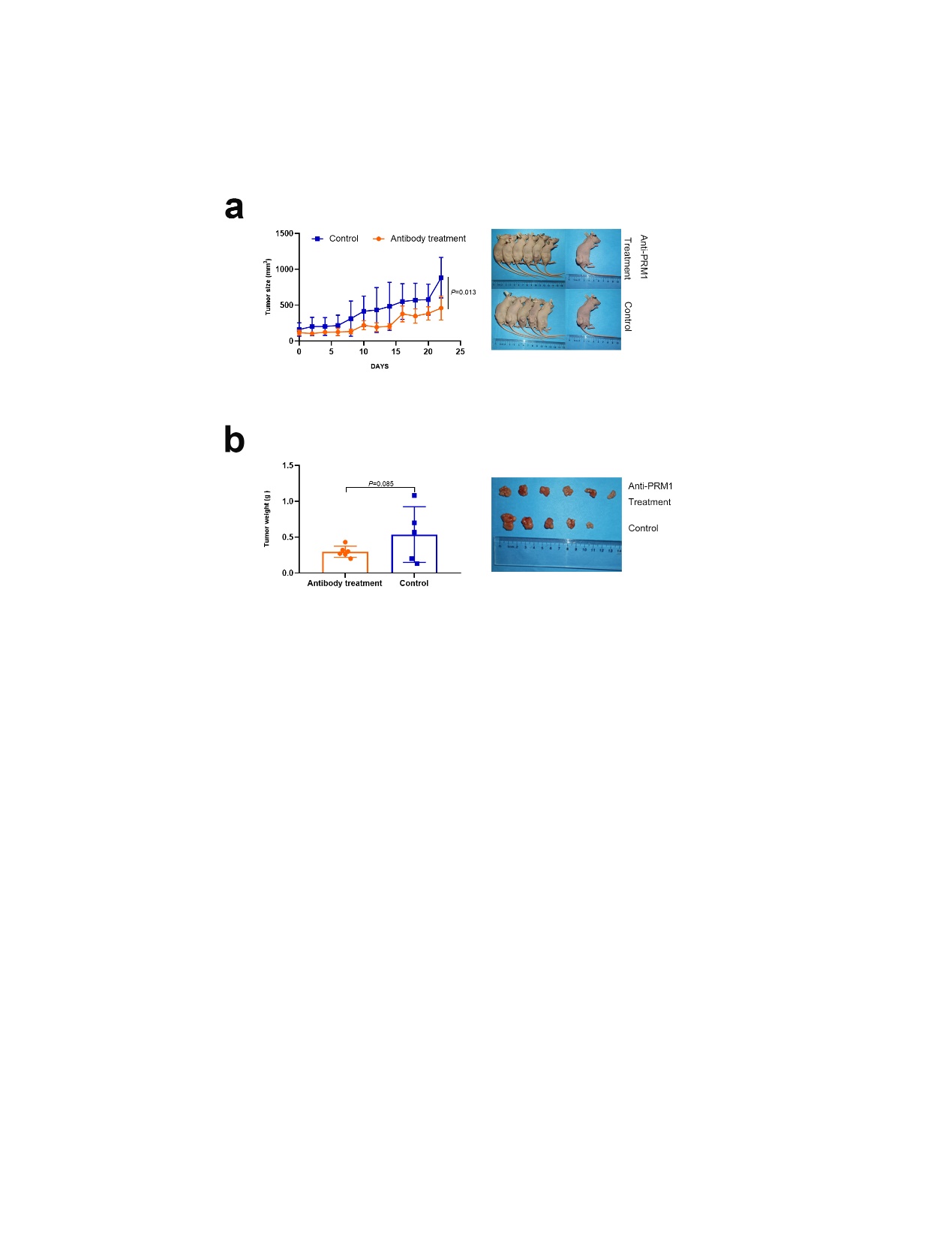


Fig. S9 Antibody against PRM1 play roles in CRC tumorigenesis. tumor growth (a) and tumor weight (b) in two groups.

**Supplementary table 1. Primers used for qRT-PCR analysis.**

| **Gene name** | **Primer sequences, 5`-3`** |
| --- | --- |
| PRM1 | F: AGAGCCGGAGCAGATATTACC |
|  | R: TCTACATCGCGGTCTGTACCT |
| GAPDH | F: CCACCCAGAAGACTGTGGAT |
|  | R: TTCTAGACGGCAGGTCAGGT |
| CDK1 | F: CATGGATTCTTCACTTGTTAAGGT |
|  | R: TCCACTTCTGGCCACACTTC |
| CDK2 | F: TGGACACGCTGCTGGATG |
|  | R: GGACTCCAAAAGCTCTGGCT |
| CDK4 | F: ATGGCTACAAGCAGATATGAG |
|  | R: TCACTCGGGGTTGCCCTC |
| CDK6 | F: GGACT TTCTTCATTCACACCG |
|  | R: GACCACTGAGGTT AGGCCA |
| CCNB1 | F: TCGCCTGAGCCTATTTTGGT |
|  | R: CAGAGAAAGCCTGACACAGGT |
| CCND1 | F: ATGGAACATCAGCTGCTGT |
|  | R: TCAGATGTCCACATCCCGC |
| CCNE1 | F: ATGGAACATCAGCTGCTGT |
|  | R: TCAGATGTCCACATCCCGC |
| MYC | F: GGACGACGAGACCTTCATCAA |
|  | R: CCAGCTTCTCTGAGACGAGCTT |
| BMI1 | F: CAAGACCAGACCACTACTGAAT |
|  | R: TATCTTCATCTGCAACCTCTCC |
| CDKN1A | F: GATGGAACTTCGACTTTGTCAC |
|  | R: GTCCACATGGTCTTCCTCTG |
| CDKN1B | F: CTAACTCTGAGGACACGCATTT |
|  | R: TTGAGTAGAAGAATCGTCGGTT |
| CDKN2A | F: GGAAGCAAATGTAGGGGTAA |
|  | R: CCAGCGTGTCCAGGAAG |
| CDKN2B | F: CCCTCGACACTCACCATGAA |
|  | R: CGACCCCTGGAATGTCACAC |
| CDKN2C | F: AAAATGGATTTGGAAGGACTGC |
|  | R: GGTCTTTCAAATCGGGATTAGC |
| CDKN2D | F: TCACACTGCTGTGGTCAGCTTT |
|  | R: AGGATGTCCACGAGGTCCTGA |
| FOXO1 | F: TACGAGTGGATGGTCAAGAG |
|  | R: ATGAACTTGCTGTGTAGGGAC |

F, Forward; R, Reverse

**Supplementary table 2.** **Antibodies and reagents**

| Reagent | Source | Identifier |
| --- | --- | --- |
| Antibodies |  |  |
| Mouse monoclonal anti-PRM1 | Briar Patch Biosciences | Mab-Hup1N；  Mab-Hup1M |
| Mouse monoclonal Anti-EGFR | Maixin | RMA-0804 |
| Mouse monoclonal Anti-VEGF | Maixin | MAB-0243 |
| Mouse monoclonal Anti-p53 | Maixin | MAB-0674 |
| Rabbit monoclonal Anti-Her-2 | Roche | 4B5 |
| mTOR (7C10) Rabbit mAb | Cell Signaling Technology | 2983 |
| Phospho-mTOR (Ser2448) (D9C2) XP ^®^ Rabbit mAb | Cell Signaling Technology | 5536 |
| Akt (pan) (C67E7) Rabbit mAb | Cell Signaling Technology | 4691 |
| Phosphor-Akt (Ser473) (D9E) XP ^®^ Rabbit mAb | Cell Signaling Technology | 4060 |
| 4E-BP1 (53H11) Rabbit mAb | Cell Signaling Technology | 9644 |
| Phosphor-4E-BP1 (Thr37/46) Antibody | Cell Signaling Technology | 9459 |
| Rabbit monoclonal Anti-Ki67 | Abcam | 16667 |
| GAPDH Mouse Monoclonal Antibody | Beyotime | AF5009 |
| β-actin Mouse Monoclonal Antibody | Beyotime | AA128 |
| Alexa fluor 488-labeled Goat Anti-Mouse IgG(H+L) | Beyotime (Shanghai, China) | A0428 |
| Synthetic protein |  |  |
| Protamine1 | Briar Patch Biosciences | Human synthetic P1 |
| SiRNAs |  |  |
| Hs_PRM1_5 | Qiagen | SI03190313 |
| Hs_PRM1_6 | Qiagen | SI04161262 |
| Hs_PRM1_7 | Qiagen | SI04136566 |
| Hs_PRM1_8 | Qiagen | SI04162151 |
| Allstars Negative Control siRNA | Qiagen | 1027280 |

**Supplementary table 3. Information for nine differentially expressed CTAs in CRC tissues**

| **Gene_short_name** | **Locus** | **Length** | **LogFC** | ***P*-value** | **Regulation** | **Description** |
| --- | --- | --- | --- | --- | --- | --- |
| ACRBP | chr12:6747240-6756626 | 9386 | 5.002241 | 0.01460 | Up | acrosin binding protein |
| TDRD6 | chr6:46620677-46703430 | 82753 | 4.681349 | 0.03813 | Up | tudor domain containing 6 |
| COX6B2 | chr19:55860673-55874628 | 13955 | 3.258219 | 0.04898 | Up | cytochrome c oxidase subunit 6B2 |
| PRM1 | chr16:11343475-11445619 | 102144 | 2.144444 | 0.01284 | Up | protamine 1 |
| POTEC | chr18:14507337-14543584 | 36247 | 4.010789 | 0.02551 | Up | POTE ankyrin domain family member C |
| KIF2C | chr1:45205489-45244451 | 38962 | 4.037632 | 0.01872 | Up | kinesin family member 2C |
| TMPRSS12 | chr12:51236702-51281667 | 44965 | 4.835296 | 0.01854 | Up | transmembrane (C-terminal) protease, serine 12 |
| IGSF11 | chr3:118619403-118864915 | 3523 | -5.34646 | 0.01335 | Down | immunoglobulin superfamily member 11 |
| RGS22 | chr8:100973163-101143496 | 4193 | -4.55205 | 0.01395 | Down | regulator of G-protein signaling 22 |

**Supplementary table 4.** **Correlations between PRM1 mRNA expression and clinicopathologic characteristics of CRC detected by qPCR**

|  | | **PRM1 mRNA** | | | |  |
| --- | --- | --- | --- | --- | --- | --- |
| **Variable** | n | **Low (%)** | **High (%)** | χ^2^ | *P*-value | |
| **Age(y)** |  |  |  | 0.032 | 0.859 | |
| ＜65 | 54 | 20(37.0%) | 34(63.0%) |  |  | |
| ≥65 | 36 | 14(38.9%) | 22(61.1%) |  |  | |
| **Gender** |  |  |  | 2.182 | 0.140 | |
| Male | 52 | 23(44.2%) | 29(55.8%) |  |  | |
| Female | 38 | 11(28.9%) | 27(71.1%) |  |  | |
| **Differentiation level** |  |  |  | 0.005 | 0.944 | |
| Well and moderately | 56 | 21(37.5%) | 35(62.5%) |  |  | |
| Poorly | 34 | 13(38.2%) | 21(61.8%) |  |  | |
| **T stage** |  |  |  | 0.151 | 0.697 | |
| 1—3 | 75 | 29(38.7%) | 46(61.3%) |  |  | |
| 4 | 15 | 5(33.3%) | 10(66.7%) |  |  | |
| **Lymph node metastasis** |  |  |  | 0.853 | 0.356 | |
| Negative | 50 | 21(42.0%) | 29(58.0%) |  |  | |
| Positive | 40 | 13(32.5%) | 27(67.5%) |  |  | |
| **Tumor size** |  |  |  | 0.750 | 0.387 | |
| ＜5cm | 38 | 16 (42.1%) | 22(57.9%) |  |  | |
| ≥5 cm | 37 | 12(32.4%) | 25(67.6%) |  |  | |
| NA | 15 |  |  |  |  | |
| **Vascular involvement** |  |  |  | 1.839 | 0.175 | |
| Negative | 45 | 20(44.4%) | 25(55.6%) |  |  | |
| Positive | 28 | 8(28.6%) | 20(71.4%) |  |  | |
| NA | 17 |  |  |  |  | |
| **Nerve involvement** |  |  |  | 3.253 | 0.071 | |
| Negative | 51 | 23(45.1%) | 28(54.9%) |  |  | |
| Positive | 22 | 5(22.7%) | 17(77.3%) |  |  | |
| NA | 17 |  |  |  |  | |
| **Clinical stage** |  |  |  | 0.853 | 0.356 | |
| Ⅰ—Ⅱ | 50 | 21(42.0%) | 29(58.0%) |  |  | |
| Ⅲ—Ⅳ | 40 | 13(32.5%) | 27(67.5%) |  |  | |
| **Tumor morphology** |  |  |  | 0.047 | 0.829 | |
| Ulcerative type | 33 | 12(36.4%) | 21(63.6%) |  |  | |
| Protrude type | 36 | 14(38.9%) | 22(61.1%) |  |  | |
| NA | 21 |  |  |  |  | |
| **EGFR** |  |  |  | No | 0.171 | |
| Negative | 18 | 9(50.0%) | 9(50.0%) |  |  | |
| Positive | 6 | 1(16.7%) | 5(83.3%) |  |  | |
| NA | 66 |  |  |  |  | |
| **p53** |  |  |  | 0.366 | 0.545 | |
| Negative | 23 | 10(43.5%) | 13(56.5%) |  |  | |
| Positive | 66 | 24(36.4%) | 42(63.6%) |  |  | |
| NA | 1 |  |  |  |  | |
| **HER-2** |  |  |  | 2.56 | 0.110 | |
| Negative | 49 | 15(30.6%) | 34(69.4%) |  |  | |
| Positive | 31 | 15(48.4%) | 16(51.6%) |  |  | |
| NA | 10 |  |  |  |  | |

**NA**: not available

**Supplementary table 5.** **Correlations between serum PRM1 and the clinicopathological characteristics of CRC in test cohort**

| **Test cohort** |  | **PRM1(pg/mL)** | | | |
| --- | --- | --- | --- | --- | --- |
| **Variable** | n | Mean ± SD | | Z | *P*-value |
| **Age (y)** |  |  |  | 0.330 | 0.741 |
| ＜65 | 54 | 814.509 ± 650.039 | |  |  |
| ≥65 | 47 | 813.485 ± 602.773 | |  |  |
| **Gender** |  |  |  | 0.566 | 0.571 |
| Male | 61 | 826.204 ± 709.790 | |  |  |
| Female | 40 | 795.470 ± 476.571 | |  |  |
| **Differentiation level** |  |  |  | 2.383 | 0.017^*^ |
| Well | 8 | 1373.329 ± 860.189 | |  |  |
| Moderately and poorly | 93 | 765.921 ± 582.455 | |  |  |
| **T stage** |  |  |  | 1.252 | 0.211 |
| 1-2 | 28 | 651.921 ± 387.142 | |  |  |
| 3-4 | 73 | 876.212 ± 687.627 | |  |  |
| **Lymph node metastasis** |  |  |  | 0.594 | 0.553 |
| Negative | 61 | 846.514 ± 660.567 | |  |  |
| Positive | 40 | 764.497 ± 572.112 | |  |  |
| **Clinical stage** |  |  |  | 1.409 | 0.159 |
| Ⅰ | 25 | 633.726 ± 392.191 | |  |  |
| Ⅱ—Ⅳ | 76 | 873.344 ± 676.629 | |  |  |
| **Tumor morphology** |  |  |  | 0.199 | 0.842 |
| Raised type | 46 | 828.847 ± 672.553 | |  |  |
| Ulcer type | 46 | 787.277 ± 614.674 | |  |  |
| NA | 9 |  |  |  |  |
| **Tumor size (cm)** |  |  |  | 0.110 | 0.912 |
| ＜5 | 49 | 778.676 ± 551.337 | |  |  |
| ≥5 | 49 | 851.102 ± 708.326 | |  |  |
| NA | 3 |  |  |  |  |
| **Vascular involvement** |  |  |  | 1.553 | 0.120 |
| Negative | 61 | 860.927 ± 657.647 | |  |  |
| Positive | 35 | 693.435 ± 567.347 | |  |  |
| NA | 5 |  |  |  |  |
| **Perineurium invasion** |  |  |  | 0.651 | 0.515 |
| Negative | 71 | 791.955 ± 651.751 | |  |  |
| Positive | 25 | 822.319 ± 568.855 | |  |  |
| NA | 5 |  |  |  |  |
| **p53** |  |  |  | 0.159 | 0.873 |
| Negative | 28 | 821.523 ± 660.513 | |  |  |
| Positive | 73 | 811.159 ± 616.069 | |  |  |
| **HER-2** |  |  |  | 0.798 | 0.425 |
| Negative | 66 | 769.475 ± 577.363 | |  |  |
| Positive | 33 | 853.214 ± 598.532 | |  |  |
| NA | 2 |  |  |  |  |

^*^ Statistically significant

**NA**: not available

| **Supplementary table 6.** **Correlations between serum PRM1 and the clinicopathological characteristics of CRC in validation cohort** | | | | | |
| --- | --- | --- | --- | --- | --- |
| **Validation cohort** | | **PRM1(pg/mL)** | | | |
| **Variable** | n | Mean ± SD | | Z | *P*-value |
| **Age (y)** |  |  |  | 0.551 | 0.582 |
| ＜65 | 99 | 547.075 ± 437.524 | |  |  |
| ≥65 | 104 | 606.458 ± 532.114 | |  |  |
| **Gender** |  |  |  | 0.357 | 0.721 |
| Male | 123 | 611.746 ± 551.352 | |  |  |
| Female | 80 | 524.841 ± 367.165 | |  |  |
| **Differentiation level** |  |  |  | 1.496 | 0.135 |
| Well and moderately | 157 | 609.726 ± 512.175 | |  |  |
| Poorly | 43 | 478.474 ± 385.959 | |  |  |
| NA | 3 |  | |  |  |
| **T stage** |  |  |  | 1.434 | 0.151 |
| 1-2 | 32 | 455.140 ± 350.319 | |  |  |
| 3-4 | 171 | 600.395 ± 507.181 | |  |  |
| **Lymph node metastasis** |  |  |  | 0.120 | 0.905 |
| Negative | 107 | 577.128 ± 480.212 | |  |  |
| Positive | 96 | 577.909 ± 499.038 | |  |  |
| **Clinical stage** |  |  |  | 1.891 | 0.059 |
| Ⅰ | 27 | 397.819 ± 271.391 | |  |  |
| Ⅱ—Ⅳ | 176 | 605.062 ± 508.119 | |  |  |
| **Tumor size (cm)** |  |  |  | 1.383 | 0.167 |
| ＜5 | 125 | 538.858 ± 452.190 | |  |  |
| ≥5 | 78 | 639.419 ± 537.697 | |  |  |
| **Vascular involvement** |  |  |  | 0.453 | 0.651 |
| Negative | 116 | 602.377 ± 529.271 | |  |  |
| Positive | 87 | 544.325 ± 427.548 | |  |  |
| **Perineurium invasion** |  |  |  | 1.290 | 0.197 |
| Negative | 136 | 584.175 ± 455.330 | |  |  |
| Positive | 66 | 559.936 ± 555.040 | |  |  |
| NA | 1 |  |  |  |  |
| **p53** |  |  |  | 3.055 | 0.002^*^ |
| Negative | 55 | 502.428 ± 578.863 | |  |  |
| Positive | 148 | 605.395 ± 448.604 | |  |  |

^*^ Statistically significant

**NA**: not available
